# In-Depth Investigation of Electrostatic Interaction-Based Hydrogel Shrinking for Volumetric Printing and Tissue Engineering Applications

**DOI:** 10.1021/acs.biomac.5c00117

**Published:** 2025-06-16

**Authors:** Dmitrii Iudin, Léon J. J. A. Gerridzen, Paulina N. Bernal, Carl C. L. Schuurmans, Myriam Neumann, Lam Nguyen, Mies J. van Steenbergen, Jaimie Hak, Wanlu Li, Cristina Casadidio, Anne Metje van Genderen, Rosalinde Masereeuw, Riccardo Levato, Yu Shrike Zhang, Bas G. P. van Ravensteijn, Tina Vermonden

**Affiliations:** Divisions of Pharmaceutics and Pharmacology Utrecht Institute for Pharmaceutical Sciences (UIPS), Science for Life, 8125 Utrecht University, 3508 TB Utrecht, The Netherlands; § Division of Engineering in Medicine, Department of Medicine, Brigham and Women’s Hospital, 1811 Harvard Medical School, Cambridge, Massachusetts 02139, United States; ∥ Department of Orthopedics, University Medical Center Utrecht, Utrecht University, 3584 CX Utrecht, The Netherlands; ⊥ Department of Clinical Sciences, Faculty of Veterinary Medicine, Utrecht University, 3584 CL Utrecht, The Netherlands; # School of Pharmacy, ChIP Research Center, 60278 University of Camerino, Via Madonna delle Carceri, 62032 Camerino, MC, Italy

## Abstract

Three-dimensional printing of hydrogels enables the fabrication of complex structures for tissue engineering. Postprinting shrinking via electrostatic interactions offers a promising strategy to better replicate the size and intricacy of native tissues. This study explores hyaluronic acid (HA)-based hydrogels that undergo shrinking upon polycation penetration and complexation focusing on the influence of the HA macromer concentration, molecular weight, cross-linking density, hydrogel initial volume, and polycation properties on shrinking efficiency. To support cell adhesion, RGD peptides were incorporated into the HA network. The polycation concentration strongly affected cell viability: a high concentration of 1 wt % resulted in reduced viability, while 0.1 wt % preserved it with effective shrinkage. Volumetrically printed structures were reduced up to 9 times in volume, achieving features as small as 42 ± 6 μm. This shrinking approach enables the fabrication of hydrogel structures with significantly reduced dimensions, making it a powerful tool for developing high-precision hydrogel structures for tissue engineering.

## Graphical abstract

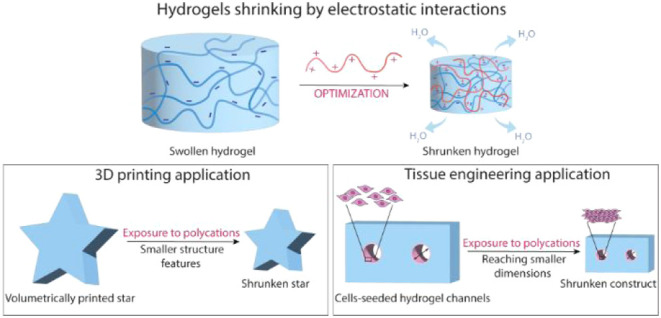

## 1 Introduction

The overarching goal of biofabrication is to create a functional (part of a) tissue or organ, which can be used as in vitro model (e.g., for drug screening) or for in vivo tissue regeneration.^1^ It has been widely reported that the functionality of cells in fabricated scaffolds depends on the structural and mechanical properties of the scaffolds such as their shape, stiffness, viscoelasticity, porosity, and surface topography.^2–6^ However, there is a lack of conclusive research to elucidate the importance of the dimensions of fabricated structures on cell functionality. The main challenge is obtaining cell-laden scaffolds containing features with dimensions closely mimicking their biological counterparts, for example, the tubular structures of the kidney nephron in the range of 10–70 μm.^7^ Currently, three-dimensional (3D) printing technologies offer exciting opportunities to create complex shapes with a high degree of design flexibility. However, when it comes to using hydrogels—often the material of choice in tissue engineering, the full potential of 3D printing is not being realized. The combination of hydrogel properties such as low stiffness and viscoelasticity makes high-resolution printing below 100 μm with good shape fidelity extremely challenging.^8^ Therefore, developing a method that can increase the resolution of printed hydrogel constructs with dimensions mimicking complicated tissue structures is of great importance.

To address the above-mentioned challenge, novel material-based approaches were developed to enhance the resolution of 3D-printed hydrogel constructs. These approaches rely on postprinting treatment of hydrogel constructs that allow to reach physiologically relevant sizes of scaffolds on demand. To this end, several triggers such as temperature changes, exposure to ions, or the use of dynamic host–guest interactions to induce hydrogel shrinking were investigated.^9–15^ Another promising approach is the use of polyelectrolytes. Typically, charged hydrogel materials can shrink on demand after the printing step by immersing the objects in a solution of oppositely charged polymers. In principle, this allows for the creation of realistic in vitro models of complex tissues such as, for example, tubular structures with physiological diameters as present in human organs.^16^ The shrinking mechanism relies on electrostatically driven complexation of the free polyelectrolytes to the oppositely charged matrix, causing expulsion of water from the hydrogel. This approach showed the potential to reduce the size of printed structures up to 10 times in volume.

However, the electrostatic interaction-based shrinking revealed limitations when applied to hydrogel-containing cells due to the cytotoxic effect of the polycations exposure. As repeatedly reported in the literature, this unfavorable effect of positively charged polymers is likely caused by the interaction with negatively charged cell membranes.^17–19^ In a previous study on the electrostatic interaction-based shrinking approach, Gong et al. used a large excess of polycations to induce efficient hydrogel shrinking which, indeed, affected cell viability.^16^ Therefore, it is crucial to elucidate what the minimal ratio between polycation and polyanion is to retain a high shrinking efficiency (overall decrease in dimensions and kinetics) and ensure good cytocompatibility.

To date, the electrostatic interaction-based shrinking approach showed versatility for use in traditional additive manufacturing techniques for hydrogel fabrication, such as extrusion-based printing with UV-curing.^16^ However, the suitability of the electrostatic shrinking method combined with light projection-based 3D printing techniques, such as volumetric printing, has not been shown yet. This technology is promising for biofabrication due to its superior ability to resolve convoluted geometries within seconds when compared to conventional extrusion-printing methods with a time scale of tens of minutes especially when producing complex structures.^20^ For instance, hydrogel structures resembling miniaturized trabecular bone were successfully volumetrically printed, as well as branched vessels, and pancreas-like models.^20–22^ Thus, the combination of volumetric printing with the shrinking approach is expected to be a promising strategy to fabricate complex structures in a fast way with an enhanced resolution.

Here, we have systematically investigated which parameters impact electrostatic interaction-based shrinkage with the ultimate goal of finding experimental conditions that simultaneously optimize shrinkage efficiency as well as cell viability. For that, hyaluronic acid (HA) modified with methacrylic groups was chosen as an anionic hydrogel matrix and poly­(2-(dimethylamino)­ethyl methacrylate) (pDMAEMA) or poly­(diallyldimethylammonium chloride) (pDADMAC) as polycations to enable the shrinking process. We present kinetic studies of polycation absorption for different polycation concentrations into the anionic hydrogel and test the cytocompatibility of kidney epithelial cells seeded into hydrogel channels under optimized conditions. We show the effect of different parameters on shrinking efficiency such as hydrogel polyanion molecular weight and its concentration, molecular weight of polycation, initial hydrogel volume, and ionic strength in phosphate-buffered saline (PBS) pH 7.4 to gain fundamental knowledge about shrinking in physiologically relevant solutions. As a final step, the application of the shrinking approach to the volumetric 3D printing technique is investigated. For that, we fabricated volumetrically printed anionic hydrogel structures and evaluated the printing resolution after the shrinking step.

## 2 Materials and Methods

### 2.1 Materials and Synthesis and Characterization Methods

#### 2.1.1 Chemicals

##### 2.1.1.1 Synthesis Reagents

Unless noted otherwise, all reagents were obtained from Sigma-Aldrich (Zwijndrecht, The Netherlands) and used as received. HA with molecular weights of 70 kDa (HA_70_), 289 kDa (HA_289_), and 1470–1530 kDa (HA_1500_) was purchased from Lifecore Biomedical (Chaska) and Contipro (Czech Republic). Maleimide-Cy3 dye from Lumiprobe (Germany) was used. The peptide with the sequence GCGYRGDSPG (RGD peptide) was purchased from Synpeptide (Shanghai, China). Triethanolamine (TEA) buffer (0.2 M, pH 8.0) was obtained from Thermo Fisher Scientific. Gelatin methacryloyl (GelMA) type B was synthesized and characterized as previously reported.^21^ All solvents were obtained from Biosolve (Valkenswaard, The Netherlands) and were dried (where indicated) using molecular sieves (4 Å) for 24 h prior to use. Demineralized water was used for reactions and buffer preparation. PBS (pH 7.4, IS 170 mM) was used (composition: 11.9 mM Na_2_HPO_4_/KH_2_PO_4_ + 137 mM NaCl + 2.7 mM KCl).

##### 2.1.1.2 Cell Culture Reagents

Dulbecco’s Modified Eagle Medium/Nutrient Mixture F-12 (DMEM-HAM/F-12, phenol red-free), Hanks’ Balanced Salt Solution (HBSS), propidium iodide (PI) and antibiotic–antimycotic solution (10^4^ units penicillin, 10 mg streptomycin, and 25 μg amphotericin B per mL, 100×) were bought from Life Technologies (Bleiswijk, The Netherlands). Calcein AM was purchased from Sanbio (Uden, The Netherlands). Fetal bovine serum (FBS) was acquired from Biowest (Nuaillé, France). 3,3′,5-Triiodo-l-thyronine sodium salt, insulin-transferrin-sodium selenite media supplement, hydrocortisone, and hEGF were acquired from Sigma-Aldrich (Zwijndrecht, The Netherlands).

#### 2.1.2 Polymer Derivatization and Synthesis

##### 2.1.2.1 Synthesis of Methacrylated HA (HAMA)

HA_70,289,1500_ were derivatized to yield HAMA by the addition of methacrylic anhydride (MA) to a HA (1–3:1 molar ratio of MA to HA’s disaccharide units for HA_70_ to obtain different degrees of methacrylation (DM; defined as the number of methacrylate groups per 100 disaccharide units), 2:1 for HA_289_ and 5:1 for HA_1500_) in water/dimethylformamide (H_2_O/DMF, volume ratio 1:1) solution according to a previously reported protocol.^16^ The DMs of HAMA_70_ samples were 12, 24, and 34 ± 3%, while DM of HAMA_289_ and HAMA_1500_ was 23 ± 2% as determined by high-performance liquid chromatography (HPLC), see Section 2.3.1.

##### 2.1.2.2 Modification of HAMA with RGD Peptide via Michael Addition

HAMA_1500_ (400 mg, 0.95 mmol repeat units, 1 equiv) was dissolved in TEA buffer (4 mg/mL) over 2 h at 37 °C. Next, the RGD peptide (80 mg, 0.08 mmol, 0.08 equiv) was dissolved in the HAMA solution, and the flask was left stirring overnight at 37 °C. The following day, the obtained reaction mixture was diluted to 1 mg/mL to decrease the viscosity of the solution to enable efficient dialysis. The dialysis was done using dialysis tubing (molecular weight cut off (MWCO) 14 kDa) against water for 4 days. The final solution was freeze-dried to obtain a fluffy product with a yield of 390 mg (87%). The degree of functionalization (DoF) was determined by size-exclusion chromatography (SEC) by comparing the HAMA-RGD UV signal at 275 nm to free RGD peptide as standards. The DoF was 5.8% (Figure S1), see Section 2.3.1.

##### 2.1.2.3 Synthesis of pDMAEMA.

pDMAEMA was synthesized by reversible addition–fragmentation chain-transfer (RAFT) polymerization using 2-(dimethylamino)­ethyl methacrylate (DMAEMA) as a monomer, azobis­(isobutyronitrile) (AIBN) as an initiator, and 4-cyano-4-[(dodecylsulfanylthiocarbonyl)­sulfanyl]­pentanoic as a chain transfer agent (CTA). The following ratio between components was used: [DMAEMA]/[AIBN]/[CTA] = 700:0.2:1. First, DMAEMA was passed through an aluminum oxide column to remove the inhibitor (hydroquinone). The purified DMAEMA (16 mL, 95 mmol, 700 equiv) was dissolved in 75 mL of dry 1,4-dioxane in a 250 mL 3-neck round-bottom flask connected to a Schlenk line. Subsequently, CTA (55 mg, 0.136 mmol, 1 equiv dissolved in 1 mL of dry dioxane) and AIBN (10 mg/mL stock solution in dry dioxane: 900 μL, 0.027 mmol, 0.2 equiv) were added to the same flask. To degas the solution, three vacuum-nitrogen flow cycles were applied. The reaction was started by submerging the flask in an oil bath at 73 °C and stirring for 15 h under a nitrogen atmosphere. Next, the reaction mixture was dialyzed for 2 days against water by using SpectraPor3 RC dialysis tubing (MWCO 3.5 kDa). The final solution was freeze-dried to obtain a dry polymer with a yield of 34%. ^1^H NMR analysis was performed to confirm the polymer structure δ: 4.1–3.9 (t, 2H, −C­(O)­O–**CH_2_**–CH_2_−), 2.6–2.5 (t, 2H, −CH_2_–**CH_2_**–N­(CH_3_)_2_−), 2.4–2.2 (s, 6H, −CH_2_–**N­(CH_3_)_2_**), 2.1–1.6 (m, 2H, −**CH_2_**–C­(CH_3_)−), 1.1–0.7 (m, 3H, −CH_2_–**C­(CH_3_)**−), see Figure S2A. Additionally, SEC revealed a *M*_w_ of 210 kDa and a polydispersity (*Đ*_SEC_) of 1.78. The chromatogram can be found in Figure S2B.

##### 2.1.2.4 Aminolysis of pDMAEMA.

The aminolysis of the CTA-containing end group was performed according to a slightly adapted protocol, published before.^23^ The synthesized pDMAEMA was weighed 3.4 g, 0.029 mmol, 1 equiv = 0.02 mmol (∼70% of all polymer chains having a CTA moiety available for the reaction as detected by UV–vis absorbance) (λ = 310 nm, Figure S3) and dissolved in 70 mL of dry tetrahydrofuran (THF) in a 100 mL round-bottom flask. Then, butylamine was dissolved (54 μL, 0.55 mmol, 28 equiv) in the reaction mixture. Finally, a few drops of tributylphosphine were added to prevent disulfide bond formation. The flask was tightly closed and the reaction mixture was left to stir at room temperature (RT) for 24 h. Subsequently, the polymer was precipitated in cold *n*-hexane, retrieved by centrifugation and decantation, and used directly for the next step.

##### 2.1.2.5 Labeling of pDMAEMA with Cy3-Maleimide.

The attachment of Cy3 was conducted based on a protocol published before.^23^ The precipitate of pDMAEMA from the previous step was dissolved in 70 mL of dry DMF in a 100 mL round-bottomed flask connected to a Schlenk line and left to stir for 24 h at RT. The solution was kept under an inert atmosphere to prevent thiol oxidation. Next, an excess of maleimide-Cy3 dye compared to the starting amount of polymer chains having a CTA moiety (25 mg, 0.038 mmol, 1.9 equiv) in 1 mL of dry DMF was added to the solution and allowed to react for 48 h at 37 °C. The reaction mixture was transferred to dialysis tubing (MWCO 3.5 kDa) and dialyzed at RT against 1 L of dimethyl sulfoxide (DMSO) with a totally three refreshments per 1 day followed by dialysis at RT against water for 1 day. The polymer was recovered by freeze-drying in a yield of 79% (2.7 g).

Next, the remainder of the noncoupled Cy3 dye was removed using a PD-10 desalting column according to supplier’s gravity protocol. For that, half of the obtained polymer (1.3 g) was dissolved in water (50 mg/mL) and passed through the column, using water as an eluent. The eluting solution was collected in 50 mL tubes and freeze-dried to obtain a pink fluffy product with an approximate yield of 85%. The UV–vis analysis showed that 7% of the polymers contain a Cy3 end group (Figure S4).

##### 2.1.2.6 Quaternization of pDMAEMA-Cy3

Quaternized pDMAEMA-Cy3 was synthesized using dimethyl sulfate (DMS) as the methylating agent adapting two previously published protocols.^24,25^ For that, a pDMAEMA-Cy3 solution (10 mg/mL) was prepared in a 1:1 v/v water/dioxane mixture. Next, a 5.3-fold excess of DMS relative to the DMAEMA repeating units was added. The reaction flask was sealed and stirred for 2 days at RT. Next, the reaction mixture was transferred to dialysis tubing (MWCO 3.5 kDa) and dialyzed against water for 2 days. The final polymer was obtained by freeze-drying with a yield of 90%. ^1^H NMR analysis was performed to confirm the polymer structure δ: 4.7–4.2 (t, 2H, −C­(O)­O–**CH**_**2**_–CH_2_−), 4.1–3.6 (t, 2H, −CH_2_–**CH**_**2**_–N­(CH_3_)_3_−) overlaps with the peak of methyl sulfate anion CH_3_SO_4_^–^ (3.7), 3.5–3.1 (s, 9H, −CH_2_–**N** ^**+**^ **(CH**_**3**_ **)**_**3**_−), 2.4–1.5 (m, 2H, −**CH**_**2**_–C­(CH_3_)−), 1.5–0.6 (m, 3H, −H_2_–**C­(CH**_**3**_ **)**−), see Figure S5.

#### 2.1.3 Characterization Methods

##### 2.1.3.1 HPLC Characterization of HAMA

The DMs of the obtained HAMA samples were determined by a reversed-phase HPLC (RF-HPLC) method developed earlier.^26^ In short, 15 mg of HAMA was dissolved in 5 mL of 0.02 M NaOH solution and incubated at 37 °C overnight to release methacrylic acid via base-catalyzed hydrolysis. Subsequently, 1 mL of a 2 M acetic acid solution was added to each sample, and the samples were injected into an Alliance Waters HPLC system equipped with UV–vis detection at 210 nm (Dual Lambda Absorbance) and a Sunfire C18 column (column temperature: 50 °C). The eluent was a mixture of acetonitrile and water 15:85 vol % (pH = 2, adjusted with perchloric acid), and the analysis was performed in an isocratic mode with a flow rate of 1 mL/min. The samples were referenced to a calibration curve of known concentrations of methacrylic acid. The obtained concentrations were then used to calculate the DM of each sample, defined as the number of methacrylate groups per 100 disaccharide units.

##### 2.1.3.2 Size-Exclusion Chromatography (SEC) Characterization of HAMA-RGD

SEC analysis was performed on a Waters Alliance System (Waters Corporation) equipped with UV (275 nm, tyrosine absorption) and differential refractive index (dRI) detectors and a PL aquagel-OH 30 column with a pore size of 8 μm (Agilent Technologies) using PBS as an eluent. Next, the calibration curve based on RGD peptide in the range of 0.05–1 mg/mL was obtained to detect the amount of RGD present in the HAMA-RGD sample (5 mg/mL) and calculate the DoF, defined as the number of RGD groups per 100 disaccharide units. All data analyses were performed using Empower 3 Software 2010 (Figure S1).

##### 2.1.3.3 ^1^H NMR Characterization of pDMAEMA and QpDMAEMA-Cy3

The synthesized pDMAEMA and QpDMAEMA-Cy3 were characterized by ^1^H NMR spectroscopy, which was performed on an Agilent 400 MR NMR spectrometer (Agilent Technologies), and chemical shifts are referred to as the residual solvent peaks CDCl_3_ (δ = 7.26 ppm) and D_2_O (δ = 4.79 ppm), respectively (Figures S2A and S5). Data analysis was performed by using MestReNova Software.

##### 2.1.3.4 SEC Characterization of pDMAEMA

The synthesized pDMAEMA was additionally characterized by SEC (Figure S2B) using a Waters Alliance System (Waters Corporation) equipped with a dRI detector and a PL aquagel-OH MIXED-M column suitable for high molecular weights (1–500 kDa) (Agilent Technologies) using 0.3 M NaAc solution, pH 4.4 as eluent. The column temperature was set to 30 °C and the flow rate was set to 1.0 mL/min. Calibrations were performed using polyethylene glycol (PEG) standards with narrow and defined molecular weights. All data analyses were performed using Empower 3 Software 2010.

##### 2.1.3.5 UV–Vis Spectroscopic Characterization of pDMAEMA-Cy3

The Cy3 coupling efficiency was estimated by UV–vis spectroscopy (Shimadzu Europe—UV-2450). In the first step, the percentage of polymers containing CTA chain ends available for modification with the dye after polymerization was assessed. For that, the absorption of the solution of pDMAEMA in THF (10.5 mg/mL) was compared to the free CTA calibration curve of 0.005–0.05 mg/mL at λ = 310 nm corresponding to the absorption of trithiocarbonate groups (Figure S3). Next, a solution of pDMAEMA-Cy3 (10 mg/mL) was prepared in DMF to determine the coupling efficiency, and the absorption was measured at λ = 555 nm (Cy3 dye). A calibration curve of Cy3 in the range of 0.001–0.01 mg/mL in DMF was obtained to determine the concentration of the dye in the polymer sample (Figure S4).

### 2.2 Hydrogel Shrinking Investigation

#### 2.2.1 Preparation of Hydrogels and Shrinking Agent Solutions

Hydrogels were prepared using HAMA_70_, HAMA_289_, and HAMA_1500_ solutions with varying polymer concentrations. Typically, HAMA was dissolved in demineralized water overnight under agitation at 4 °C. The polymer solution was supplemented with 0.1 wt % Irgacure 2959 prior to UV cross-linking. The resulting polymer solutions were cast in various cylindrical polydimethylsiloxane (PDMS) molds (for a typical experiment: diameter = 4.5 cm, height = 2 mm) and subsequently UV-irradiated (Bluepoint UV source, 320–500 nm, the distance between the light source and the sample was set to 5 cm, lamp intensity was 305 mW/cm^2^ at the sample location) for 2 min (methacrylate conversion for HAMA gels was determined by RF-HPLC method to be >95% following a similar procedure as described above in the section “HPLC characterization of HAMA”). Subsequently, a biopsy punch (Integra Miltex, various diameters) was used to cut out cylindrical hydrogels of certain diameters and heights. The hydrogels used for standard measurements were 6 mm in diameter (*d*) and 2 mm in height (*h*), yielding an initial volume of ∼57 mm^3^. The hydrogels were directly used without any other treatment. Additionally, cylindrical gels with dimensions of *d* = 8 mm, *h* = 2.7 mm (135 mm^3^) and *d* = 12 mm, *h* = 4 mm (450 mm^3^) were prepared following a similar procedure to investigate the effect of hydrogel size on the shrinkage process.

Shrinking agents (synthesized (Q)­pDMAEMA-Cy3 and purchased pDADMAC with molecular weight ranges of <100, 200–350, and 400–500 kDa) were dissolved overnight in phosphate-buffered saline (ionic strength = 170 mM, pH 7.4) in the range of 0.02–2.0 wt %.

#### 2.2.2 Shrinking Kinetics and Efficiency

Shrinking efficiency (volumetric shrinking factor *V*_0_/*V*_s_ with *V*_0_ defined as the starting volume and *V*_s_ as the volume (in mm^3^) after shrinking at time *t*) was investigated by varying the following parameters of the shrinking system: initial polycation (A) or polyanion concentration (B), polycation molecular weight (C) and initial hydrogel volume (D) as presented in Table 1.

**Table 1:** Overview of Experimental Parameters to Assess Their Influence on Shrinking Efficiency

	polycation type	polycation, wt %	polyanion conc, wt %	polycation molecular weight, kDa	hydrogel *V*_0_, mm^3^
A	pDMAEMA	0.02–0.15	1	210	57
B	pDADMAC	1	1, 2.5, 5 or 8	400–500	57
C	pDADMAC	1	2.5	<100, 200–350 or 400–500	57
D	pDMAEMA	0.1	1	210	57, 135 or 450

In a typical experiment, the HAMA hydrogels (*n* = 3) were incubated in a 2 wt % pDADMAC solution (2 mL) at RT. For that, 1–2.5 wt % HAMA hydrogels were prepared with HAMA_1500_ and 5–8 wt % hydrogels with HAMA_70_. The HAMA molecular weight was adapted for each wt % to circumvent solution inhomogeneities and excessive increase in viscosity observed when attempting to prepare higher wt % solutions of high molecular weight HA’s.

In contrast to the typical procedure, the shrinking efficiency depending on the initial hydrogel volume (D) was investigated upon exposure to pDMAEMA-Cy3 solutions (0.1 wt %; 2, 4.8, and 16 mL) to visualize polycation uptake. Solution volumes were adjusted in accordance with the initial hydrogel volumes (57, 135, and 450 mm^3^) to keep the charge ratio during the shrinking constant. The polycation absorption for this experiment was monitored, as described below.

Finally, the hydrogel diameter and height for all samples were measured to track the volumetric shrinkage in time. Comparing the shrinking efficiency was done when the hydrogel volumes plateaued and did not change volume over a 24 h time-period.

To determine the dependence of the shrinking kinetics on polycation concentration (volumetric shrinking factor as a function of time), HAMA-based hydrogels (HAMA_1500_, DM 23%, 1 wt %) were prepared. Next, solutions of (Q)­pDMAEMA-Cy3 were used as shrinking agents in a concentration range of 0.02–0.15 wt %. The hydrogels (*n* = 3) were incubated at RT in 2 mL of a polycation solution with the predetermined concentration, and their diameters and heights were monitored using Vernier calipers over time to track the volumetric shrinkage.

Additionally, polycation uptake was analyzed over time. To this end, the fluorescence of 2 mL of a supernatant at different time points was monitored on a Jasco spectrofluorometer with a cuvette holder (FP-8300) to detect a fluorescent signal reduction (λ_ex_ = 545 nm, λ_em_ = 570 nm) with adding this volume back to samples for further polycation absorption. The absorbed amounts were determined according to a calibration curve based on polymer dilutions with predetermined concentrations (Figure S6).

### 2.3 Cell Viability Assay of the Shrinking Process

Conditionally immortalized proximal tubular epithelial cells (ciPTECs 21.2), purchased from Cell4Pharma (Oss, The Netherlands), used throughout the study were cultured in accordance with a previously published procedure.^27^ Cytocompatibility of the shrinking process was evaluated using hydrogels based on a 1 wt % HAMA-RGD composition that was cross-linked with the use of lithium phenyl-2,4,6-trimethylbenzoylphosphinate (LAP). Hydrogels were cast in the plastic mold with inserted 27G needles to form three parallel channels per gel (Figure S7). Then, ciPTECs were seeded inside each channel by flushing it with 10^7^ cells/mL of culture medium. The hydrogels were placed in a 33 °C incubator with subsequent 180° flipping once after 2 h to ensure a more homogeneous distribution of cells. After 2 days, the prepared hydrogels with cells were divided into two groups and placed in parallel in two solutions of pDMAEMA in culture medium with concentrations of 1 and 0.1 wt % (5 mL per gel) that represent a 10× excess and the minimum amount of polycation needed for effective shrinking, respectively. Additionally, a control group of hydrogels was placed in a polycation-free medium. The medium additionally contained 2 vol % antibiotic–antimycotic to prevent contaminations. Hydrogels were exposed to polycation solutions for 24 h for both polycation concentrations. After this 24 h period, the gels were placed in a polycation-free culture medium. Over the first 8 days, the hydrogels were kept at 33 °C for the proliferation stage and moved for another 6 days to 37 °C to induce maturation. The temperature sensitivity for the proliferation and maturation stages is related to the presence of the SV40T-large antigen.^28^ At various time points (1, 5, 8, and 14 days) samples were subjected to live–dead staining where living cells were stained with calcein AM (25 μL/mL, revealing calcein after hydrolysis, green) and dead cells with propidium iodide (50 μL/mL, penetrates only dead cells and binds to DNA, red) with an incubation time of 15 min at 33 °C. The samples were imaged using confocal microscopy (Leica Stellaris 8 with 10× magnification).

### 2.4 Volumetric Printing

Volumetric printing was carried out using a tomographic volumetric printer (Tomolite, Readily3D, Lausanne, Switzerland).^21^ Specifically, resins were made from 1.0 wt % HAMA_1500_ or 4.0 wt % GelMA B solution, both supplemented with 0.1 wt % LAP as photoinitiator, which is suitable to be initiated with the 405 nm laser. Similar shrinking factors were observed regardless of whether Irgacure 2959 or LAP was used as an initiator in comparison experiments. Resins were placed in borosilicate glass vials with an 18 mm inner diameter. For HAMA, the high viscosity of the polymer prevented print sedimentation, and GelMA samples were thermally gelated at 4 °C to prevent the formation of sedimentation throughout the printing process. Briefly, volumetric printing was induced by a laser beam at 405 nm directed onto a digital micromirror device (DMD), which was modulated into tomographically filtered back projections delivered onto the resin-filled vials. The projections were calculated using commercial software (Apparite, Readily3D, Switzerland). The average light intensity before the printing container was 7.89 mW/cm^2^ during printing. HAMA and GelMA samples were exposed to light doses of 625 mJ/cm^2^ (exposure time = 79.2 s) and 175 mJ/cm^2^ (exposure time = 22.1 s), respectively. Samples were subsequently washed with PBS to remove un-cross-linked polymer and excess photoinitiator. For HAMA-based solutions, 0.002 wt % (2,2,6,6-tetramethylpiperidin-1-yl)­oxyl (TEMPO) was added as a radical inhibitor to increase printing resolution. Washed samples were postcured through UV irradiation for 10 min (λ = 365 nm; CL-1000L UV Cross-linker, UVP, U.K.) while immersed in 0.1 wt % LAP solution. Samples were either stained with Safranin-O for 2 min to facilitate microscopic imaging or used without further staining.

Volumetrically printed, star-shaped hydrogels were subsequently imaged and measured using a stereomicroscope (Olympus SZ61 coupled with an Olympus DP70 digital camera; Olympus Soft Imaging Solutions GmbH, The Netherlands) and shrunken. After being shrunk, the printed shrunken hydrogels were again imaged and measured via stereomicroscopy. To measure the star tip widths, straight lines were drawn along each side of each tip and the width was determined as the length at which the print overlapped with the drawn lines (Figure S8).

For volume measurements of the printed gyroidal samples, microcomputed tomography (μCT) was performed at a voxel size of 15 μm^3^ using 90 kV tube voltage, 200 μA tube current, and 26 s of scan time. Scanning was performed using a Quantum FX μCT scanner (PerkinElmer) and the respective software (Quantum FX μCT software, PerkinElmer). 3D reconstruction was carried out automatically after completion of each scan. To image constructs immersed in water and prevent collapse of the finer structures present in the gyroids, these samples were first incubated in 20 mM Hexabrix 320 (Guerbet, France) overnight at 37 °C to act as a contrast agent and enable imaging of the printed structures, washed in PBS and scanned in a water-filled reservoir. The volume of the resulting scans was calculated using the “volume fraction” function of the Bone J plugin for ImageJ ().

### 2.5 Statistical Analysis

Unless noted otherwise, all experiments were done in technical replicates, and the mean value ± standard deviation (SD) was calculated for each data point. Statistical analysis where indicated was performed in GraphPad PRISM 10 (GraphPad Software Inc., CA), using (multiple) unpaired *t* test. The following symbols were used to assign statistical significance depending on the resulting *p*-value: ns (not significant) indicates *p* > 0.05, *p ≤ 0.05, **p ≤ 0.01, ***p ≤ 0.001, and ****p ≤ 0.0001.

## 3 Results and Discussions

To find optimal shrinking conditions for the electrostatic interaction-based approach, HA_1500_ was selected to prepare the hydrogel matrix. HA is a highly negatively charged polymer under physiological conditions, which is present in the natural extracellular matrix and, therefore, generally, exhibits good cytocompatibility.^29,30^ We modified HA with methacrylic groups using MA according to a previously published procedure (DM = 23 ± 2%) to enable photo-cross-linking and obtain mechanically robust hydrogels (Figure 1A).^16^ pDADMAC and pDMAEMA (see chemical structures in Figure 1B) were chosen as polycations to induce shrinking as they both exhibit high water solubility at pH 7.4 and can be purchased or synthesized with a wide range of molecular weights required for this study.^31,32^ The mechanism of shrinking relies on electrostatic interactions between negatively charged hydrogels and polycations. These interactions lead to the release of their counterions, the formation of charge-neutral complexes, and an increase in hydrophobicity, promoting water expulsion, that, in turn, results in hydrogel shrinkage (Figure 1C). An example of the hydrogel size changes after 48 h when equilibrium shrinking was reached is presented in Figure 1D.

**Figure 1:**
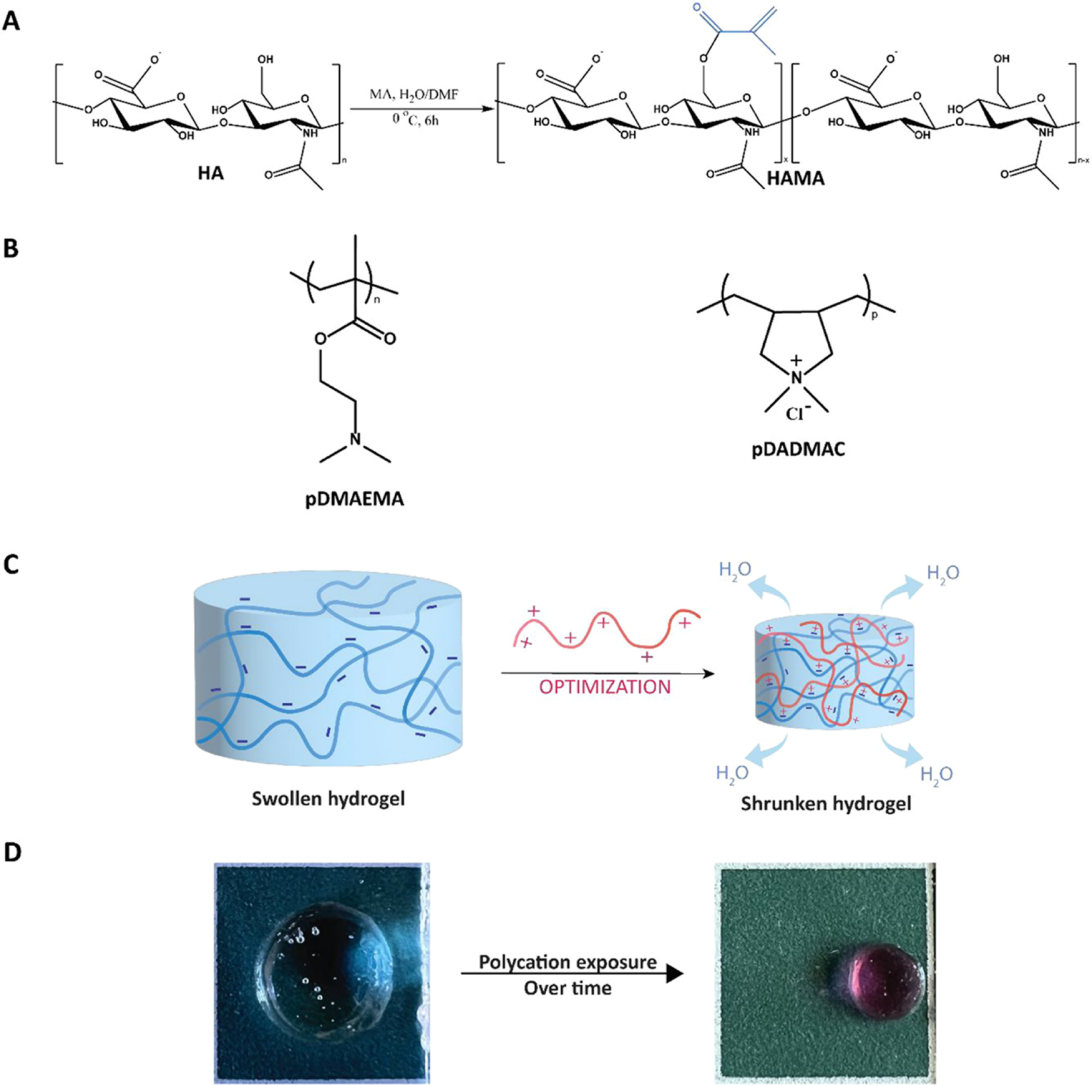
(A) HAMA synthesis scheme. (B) Chemical structures of pDMAEMA and PDADMAC. (C) Schematic illustration of the electrostatic interaction-based shrinking mechanism. (D) 1 wt % HAMA hydrogel before and after exposure to 6.4 μmol/mL pDMAEMA-Cy3 for 48 h (scale of the square is 100 mm^2^).

To verify that the hydrogel shrinking is driven by electrostatic interactions, the effect of ionic strength on hydrogel–polycation interaction and hydrogel shrinking was studied for HAMA hydrogels incubated in a solution of pDADMAC 400–500 kDa (Figure S9). It was shown that with increasing ionic strength from 17 to 680 mM, the shrinking of the HAMA hydrogel decreased. This dependency of hydrogel shrinking on ionic strength is due to the increased screening of charges of polymers by ionic solutes that occurs at higher ionic strengths that, in turn, demonstrates the electrostatic nature of hydrogel shrinking.^33–35^ As expected, screening of the charges present on both the polycation and the hydrogel network reduces the electrostatic polycation–hydrogel interactions and results in reduced water expulsion from hydrogels. Previously, it was demonstrated for the presented shrinking approach that the increased concentration of counterions can also induce shrinking alone. However, the role of counterions in shrinking is significantly lower (and reversible) as compared to the long-lasting shrinking effect induced by polycations.^16^

### 3.1 Shrinking Kinetics and Efficiency—Effect of Different Parameters

Based on the proof-of-concept of the shrinking mechanism, next, we set out to systematically elucidate which experimental parameters impact the electrostatic interaction-based shrinkage under physiological conditions to obtain optimal parameters to improve its performance for cell-laden hydrogels. First, we investigated the effect of polyanion macromer concentration, its molecular weight and cross-linking density, as well as polycation molecular weight, parameters that can directly affect shrinking kinetics and efficiency. Subsequently, we investigated the effect of polycation concentration as a major parameter affecting not only the shrinking process but also cell viability. Additionally, we evaluated the applicability of the shrinking approach for hydrogels with different initial volumes.

#### 3.1.1 Molecular Weight of Polyanion (Hydrogel Macromer), Its Degree of Methacrylation and Concentration

First, the effect of the molecular weight of polyanion on the shrinking efficiency was investigated by preparing 1 wt % hydrogels composed of HAMA with three different molecular weights, namely 70, 289, and 1470–1530 kDa (Figure S10). We observed that increasing the molecular weight of the polyanions used to produce the hydrogels did not affect the hydrogel shrinking. This lack of effect is likely due to the cross-linking step performed during hydrogel fabrication, which links all macromers together into a large anionic network. In such a network, the individual HAMA chains are no longer distinguishable, eliminating any potential effect of this parameter when the weight percentage of HAMA is fixed. Similarly, changing the methacrylation degree in the range of 12–34% (and thus the network cross-linking density) did not impact the hydrogel shrinkage efficiency (Figure S11). Likely, at the low HAMA concentration (1 wt %), even at high cross-link density, changes in network stiffness and/or pore size are apparently not affecting polycation uptake by the hydrogel network and effective shrinkage.

Next, the effect of the polyanion concentration on the hydrogel shrinking efficiency was studied. To this end, hydrogels with four different HAMA contents, namely 1, 2.5, 5, and 8 wt %, were prepared (Figure 2A). We observed that the hydrogel shrinking efficiency was highly dependent on the initial HAMA weight percentage. Hydrogels with a high initial polyanion content (8 wt %) showed no shrinkage as compared to hydrogels made with a low content (1 wt %) demonstrating a shrinking factor up to 6.5 times. A possible explanation is that by increasing the initial macromer content of the hydrogel, its initial water content is lower and its cross-link density is increased, which might impair polycation penetration and/or make the hydrogel network more rigid and thus more resistant against shrinkage.

**Figure 2:**
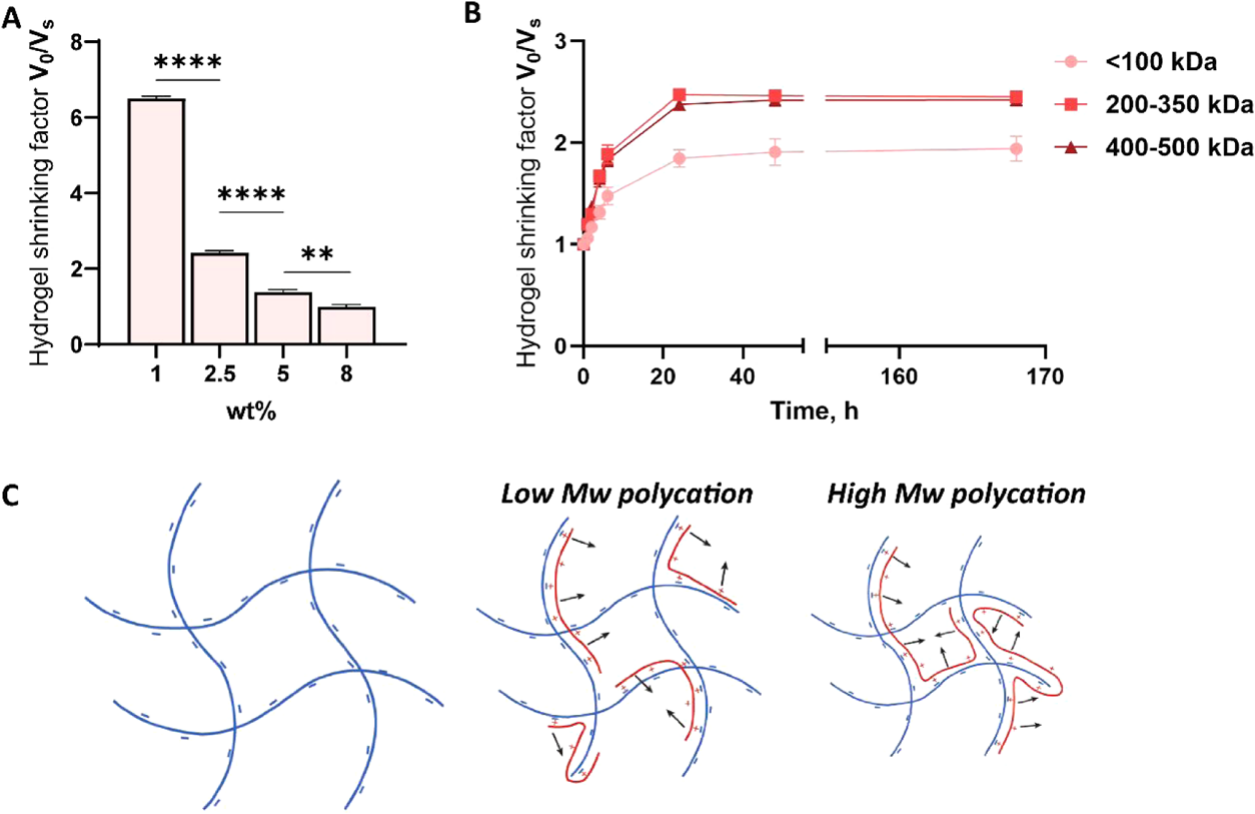
(A) Shrinking factors for HAMA_70_ (5–8 wt %) and HAMA_1500_ (1–2.5 wt %) hydrogels after incubation in a 2.0 wt % pDADMAC solution (molecular weight: 400–500 kDa). Statistical analysis (unpaired *t* test): **p ≤ 0.01 and ****p ≤ 0.0001. (B) Shrinking factors change over time for HAMA_1500_ hydrogels (2.5 wt %) upon incubation with 2 wt % pDADMAC with an average molecular weight of <100, 200–350, and 400–500 kDa. The difference in shrinking factors between <100 kDa and other two molecular weights is significant. The difference between 200–350 and 400–500 kDa is ns (*p* > 0.05). (C) Interaction between the anionic hydrogel network and polycations of different molecular weights. All measurements were performed after the shrinking equilibrium was reached (RT, pH 7.4, 170 mM of ionic strength). Initial hydrogel volume was 57 mm^3^. Data are shown as the average of 3 samples ± SD, for some time points deviation, the error bars are too small to be visible.

Next, to elucidate which of these two contributions is causing a decreased shrinking efficiency with increasing initial polymer content, we conducted an additional experiment using pDMAEMA-Cy3 (*M*_w_ of 207 kDa, see synthesis discussion in Section 3.1.3) and 8 wt % hydrogels. We placed the hydrogels in 0.8 wt % polycation solution to monitor the polycation uptake (spectrofluorometric analysis and taking macroscopic pictures) and compared the results with volumetric changes of the hydrogels. The results (Figure S12) showed that during 3 weeks of monitoring, the hydrogel demonstrated no measurable shrinking, while the polycation uptake reached the theoretically predicated point of charge neutralization. Moreover, the polycation distribution was macroscopically homogeneous. Based on these findings, we could conclude that the main limiting factor to obtaining significant shrinkage for the high wt % hydrogels is increased network rigidity and not a hampered polycation uptake.

Based on the results above, we established that HA molecular weight and DM do not affect shrinking for 1 wt % hydrogels, while an increase in initial solid content drastically deteriorated shrinking properties. Hence, the hydrogel matrix comprising HAMA_1500_ (DM 23%) was selected as the reference system for the subsequent experiments.

#### 3.1.2 Molecular Weight of the Polycation

The effect of the polycation’s molecular weight on hydrogel shrinking efficiency was also studied. Here, 2.5 wt % hydrogels were shrunken in 2 wt % pDADMAC solutions (substantial excess of polycations to ensure the highest attainable shrinkage) of three molecular weight ranges (<100, 200–350, and 400–500 kDa). The choice of the initial hydrogel wt % was based on the hypothesis that a hydrogel mesh size can affect the uptake of polycations with different molecular weights. Based on Young’s moduli of the hydrogels reported previously, we estimated that the initial mesh size of 2.5 wt % hydrogels is smaller (approximately 8 nm) in comparison with 1 wt % condition, with a mesh size of approximately 15 nm.^16,36^ However, in contrast to our initial hypothesis, we observed that increasing the molecular weight of the polycation leads to more hydrogel dehydration and shrinkage. Clearly, there is no hindering effect of the mesh size on the efficiency of polycation uptake regardless of their molecular weight. Moreover, a molecular weight of the polycation above 200 kDa does not contribute any further to the final shrinking factors (Figure 2B). This finding can be rationalized by the fact that compared with shorter chains, higher molecular weight polymers can more easily bridge the mesh size of the hydrogel network, thereby generating more binding locations that result in greater network contraction (Figure 2C).

Although shorter polycations are probably more likely to bind reversibly to the negatively charged network, the dissociation of the shortest polycations used here is highly unlikely. Theoretical estimations indicate that the strength of the electrostatic interactions between the polycations and the polyanion hydrogel network is higher than 30 *k*_B_ *T*, regardless of the molecular weight ranges tested, which is sufficient to prevent polyelectrolytes dissociation at low salt concentrations (Figure S13).^37^ Therefore, the observed differences in shrinkage behavior cannot be attributed to incomplete charge neutralization.

To conclude, polycations with a molecular weight of at least 200 kDa are required to maximize the shrinkage. Therefore, these higher molecular weight polycations were used for further optimization of the shrinking process.

#### 3.1.3 Polycation Concentration

pDMAEMA synthesized by RAFT polymerization with *M*_w_ of 207 kDa (*Đ*_SEC_ = 1.78) was selected as a polycationic shrinking agent to study the effect of polycation concentration on shrinking efficiency, as a major parameter affecting cell viability (Figures 3A and S2A,B). This choice was based on a straightforward polymer synthesis route, which allows end-capping of the polymer chains with a fluorescent Cy3 label. To achieve this, terminal trithiocarbonates originating from the CTA used during the RAFT polymerization were converted into thiols by aminolysis. Subsequently, these thiols reacted with a maleimide-functionalized Cy3 dye, resulting in 7% of the polymers having a fluorescent label (Figures 3B and S3, S4).

**Figure 3:**
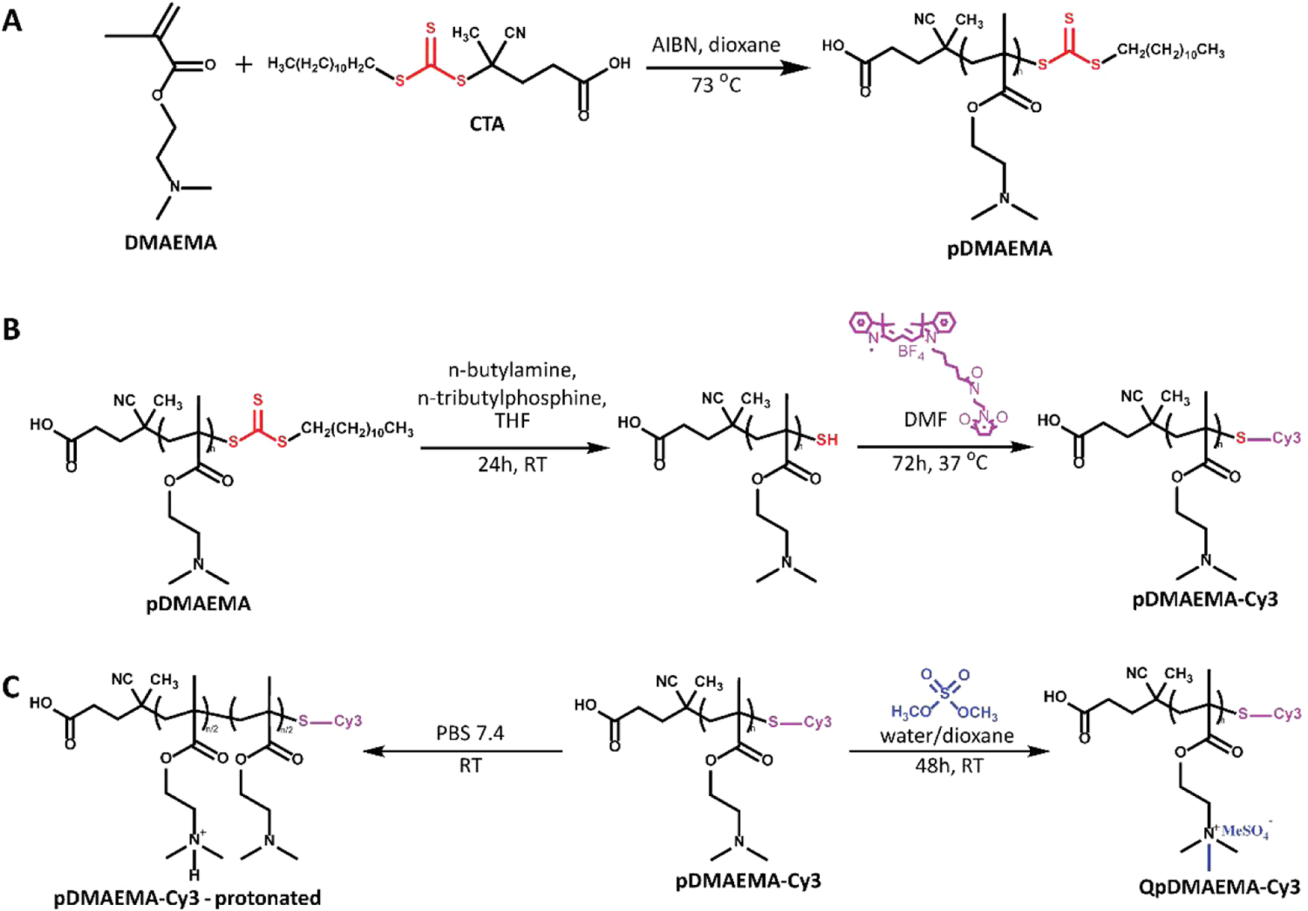
(A) Synthesis of pDMAEMA was by RAFT polymerization. (B) Attachment of cyanine3 dye to pDMAEMA. (C) Polycation charge formation occurs via protonation (left) or quaternization (right).

Based on the results of the experiment with altering ionic strength discussed at the beginning of the results and discussions section (Figure S9), we elucidated the important relationship between the charge density of polymers of the system and the shrinking efficiency. Therefore, to acquire more insights into this parameter, we compared two different polycation charge densities and the effect of a permanent vs pH-dependent charge on shrinking while fixing pH at a physiologically relevant value of 7.4. For that, two ways of charge formation were chosen. First, the synthesized pDMAEMA was used directly as a weak polyelectrolyte and dissolved in PBS buffer (pH 7.4, IS = 170–180 mM, adjusted with several drops of 4 M HCl) resulting in protonation of approximately 50% of all repeating units (p*K*_a_ = 7.5).^38^ Second, pDMAEMA was quaternized to obtain a strong polyelectrolyte (QpDMAEMA) containing pH-independent cationic groups. Quantitative quaternization was confirmed by ^1^H NMR (Figures 3C and S5), resulting in a polyelectrolyte in which each repeat unit carries a cationic charge. Then, 1 wt % HAMA-based hydrogels were placed in the solutions of polycations with different concentrations to investigate the shrinking kinetics. The fluorescent labeling of these polycations allowed monitoring of the polymer absorption by measuring the reduction of the fluorescent signal of the supernatants over time.

For these experiments, we expressed the polycation concentrations as a number of moles of monomer units (*n*_monomer units_). This value is directly related to the amount of positive charges present in the solution. For pDMAEMA the amount of positive charges is approximately *n*_monomer units_/2 due to a degree of protonation of approximately 50% at pH 7.4.^38^ For QpDMAEMA the amounts of charges and monomer units are equal. The calculation of the amount of monomer units is based on:

$$n_{\mathrm{monomer}\;\mathrm{unit}},\mathrm{\mu mol}/\mathrm{mL}=\frac{C_{\mathrm{polymer}},g/\mathrm{mL}}{M_{\mathrm{monomer}},g/\mathrm{\mu mol}}$$

Then, taking into consideration that HAMA hydrogels contain 1.3 μmol of monomer units per gel, we calculated the initial molar monomer unit ratios between polycations and polyanion. We used the following polycation/polyanion ratios: 9.5 μmol/mL (15:1), 6.4 μmol/mL (10:1), 3.2 μmol/mL (5:1), and 1.3 μmol/mL (2:1), keeping the total volume of polycation solution at 2 mL. As shown in Figure 4A, when we initiated shrinking using pDMAEMA with the starting ratio of 15:1 and 10:1, we observed complete shrinkage within 48 h, and shrinking factors (*V*_0_/*V*_s_) of approximately 7 were achieved under these conditions. Upon lower starting ratios, the shrinking occurs slower for the intermediate ratio 5:1, while the lowest ratio 2:1 hardly showed shrinking even after 168 h. In comparison, HAMA hydrogels exposed to QpDMAEMA (Figure 4C) show continuous shrinking up to a shrinking factor of 9 over a week. This finding can be rationalized by the hypothesis that QpDMAEMA can compensate for more negative charges of the HAMA network due to a higher amount of positive charges available if the molar polycation absorption by hydrogels is the same.

**Figure 4:**
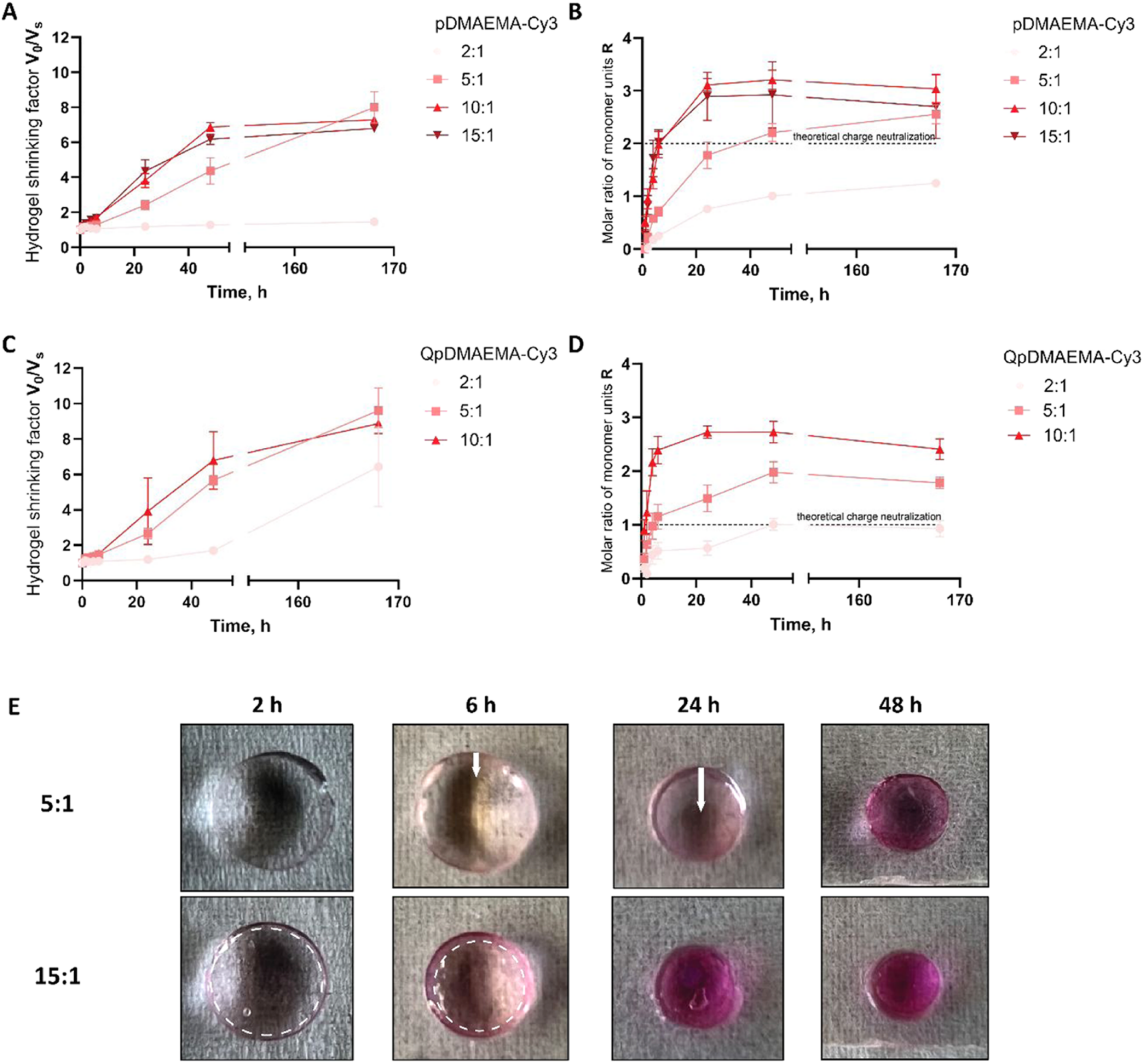
Shrinking factors over time for 1 wt % HAMA_1500_ hydrogels with *V*_0_ = 57 mm^3^ treated with (A) pDMAEMA-Cy3 and (C) QpDMAEMA-Cy3. Analysis of the absorbed amounts of polycations is presented as the molar ratio of monomer units (*R*) of the polycation and HAMA for (B) pDMAEMA-Cy3 and (D) QpDMAEMA-Cy3. The initial molar ratios of monomer units of the polycation and polyanion 2:1, 5:1, 10:1, and 15:1 correspond to concentrations 1.3, 3.2, 6.4, and 9.5 μmol/mL, respectively. Data are shown as the average of 3 samples ± SD, for some data points, the error bars are too small to be visible, (E) macroscopic pictures of pDMAEMA-Cy3 absorption over time for two ratios (5:1 and 15:1) into a 1 wt % HAMA_1500_ hydrogel at pH 7.4, ionic strength: 170–180 mM.

To investigate if the above-mentioned hypothesis is true, we analyzed the absorbed amounts of polycation during the shrinking process over time presented as the molar ratio of monomer units of polycation/polyanion in the gel (Figure 4B,D). The observed absorption kinetics are similar for both pDMAEMA and its quaternized equivalent. Based on this, we conclude that the polycation uptake is likely independent of the initial positive charge density. The findings described above are in accordance with literature reports for negatively charged microgels based on acrylic acid and positively charged peptides.^39^ The authors observed that the peptide uptake was similar regardless of the initial positive charge density of the peptides while the shrinking efficiency was increasing with increasing charge density. Additionally, upon reaching the highest achievable shrinking factor (at a starting ratio of 5:1 or higher for both polycations), hydrogels shrunken with QpDMAEMA contained a relatively higher amount of positive charges. The final charge ratio was between 1.8:1 and 2.5:1, as shown in Figure 2D. While for pDMAEMA the charge ratio remained below 1.5:1. Note that because of the ∼50% degree of ionization of pDMAEMA the monomer unit ratio obtained during the experiment (≤3:1) should be divided by a factor of 2 to achieve the actual charge ratio (Figure 4B). As a result, these hydrogels contain less positively charged moieties in comparison with their quaternized equivalent.

To investigate the relation between shrinking kinetics and the rate of polycation absorption, we compared both the shrinking and absorption kinetics. Over 95% of the polycation uptake for the initial charge ratios above 10:1 happened within the first 24 h, while the volumetric shrinking factors remained ≤4 and reached their final values of 7–9 only within a week. Hence, the polycation absorption is significantly faster than that of the hydrogel shrinking process. This may result from a prolonged reorganization of the polycation chains, which over time were able to compensate more negative charges, leading to stronger pulling forces.

Additionally, the absorption of pDMAEMA-Cy3 (initial ratios 5:1 and 15:1) could be readily visualized macroscopically over time as the labeled polymer has an intense pink color (Figure 4E). At high polycation concentration (ratio 15:1), the absorption starts by the formation of a well-defined moving front that reaches a homogeneous distribution over time (see white dashed circles). In comparison, for the lower polycation concentration (ratio 5:1), the moving front was blurred, likely due to a slower inward diffusion of the polycations that coincides with the slower shrinking kinetics (see white arrows). The obtained results are in agreement with the data presented in the literature.^16,40–43^ These papers demonstrated the formation of a saturated moving front for positively charged lysozyme or quaternized chitosan absorbed by negatively charged poly­(acrylic acid), poly­(styrenesulfonate), or hyaluronic acid-based gels.

As a result, we elucidated that the initial charge ratio of approximately 2.5:1 is sufficient to yield a high shrinking efficiency of 7–9 for both pDMAEMA and its quaternized equivalent. This value is at least 10× lower than was used in previous work on electrostatic interaction-based hydrogel shrinking for cytocompatibility studies, making these optimized conditions likely more cytocompatible (see Section 3.2).

#### 3.1.4 Initial Hydrogel Volume

Next, the effect of the initial hydrogel volume on the shrinking factor and kinetics was investigated to evaluate whether the process remains efficient for objects of different sizes. For this, cylindrical HAMA hydrogels with initial volumes of 57, 135, and 450 mm^3^ were placed in a solution of pDMAEMA-Cy3 of different volumes as described in Section 2.4.2. Adjusting the polycation solution volume was necessary to keep the initial overall charge ratio in the system equal.

We observed that the higher the initial hydrogel volume, the more time it took to reach the equilibrium shrunken state. In particular, 57 mm^3^ hydrogels reached a plateau in shrinking factor after 48 h and 135 mm^3^ samples after 336 h with a shrinking factor of approximately 7.5. However, the shrinking curve for the 450 mm^3^ hydrogels did not show a plateau even after 504 h (Figure 5A). The size changes of the hydrogels with initial volumes of 135 and 450 mm^3^ are shown in Figure 5C together with the macroscopic appearance of their cross sections to demonstrate the polycation distribution over the hydrogels. Even though macroscopically we observed a homogeneous distribution of polycation for both hydrogels, the postponed shrinking of the 450 mm^3^ sample can be rationalized by possible inhomogeneities on a microscopic scale that lead to incomplete charge compensation.

**Figure 5:**
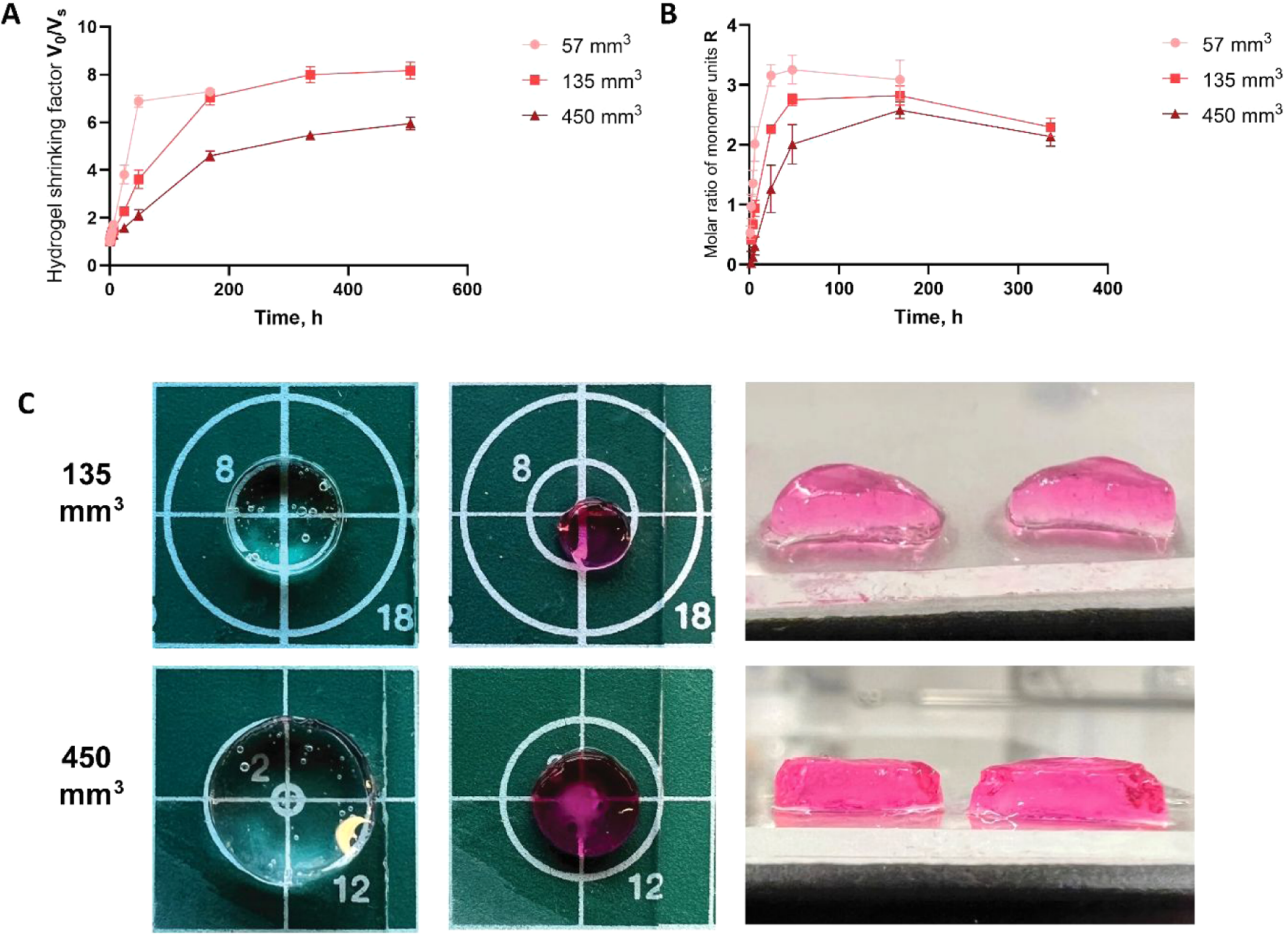
(A) Shrinking factors over time for 1 wt % HAMA_1500_ hydrogels of different initial volumes 57, 135, and 450 mm^3^, respectively. All measurements were done at RT, pH 7.4, and an ionic strength of 170–180 mM. Hydrogels were placed in a solution of 0.1 wt % pDMAEMA-Cy3 (6.4 μmol/mL of monomer units) of different volumes (2, 4.8, and 16 mL). (B) Absorbed amounts of polycations are presented as the molar ratio of monomer units (*R*) of polycation and HAMA for different initial volumes of hydrogels. The difference in absorption kinetics between various initial hydrogel volumes is significant over the first 48 h. Starting from 168 h the difference is ns. (C) 1 wt % HAMA_1500_-based hydrogels of two different initial hydrogel volumes 135 and 450 mm^3^ before and after shrinking at time point 3 weeks. For the 135 mm^3^ sample, the diameter of the inner white circle is 8 mm. For the 450 mm^3^ sample, the diameter of the outer white circle is 12 mm. Additionally, the cross sections of both hydrogels after cutting show the homogeneous polycation distribution. Data are shown as the average of 3 samples (57 mm^3^) and 2 samples (135, 400 mm^3^) ± SD, for some time points the error bars are too small to be visible.

The trend in the shrinking kinetics is directly related to the different rates of polycation absorption. The polycation absorption showed a significant dependence on the initial hydrogel volume over the first 48 h. However, after 168 h (1 week), all tested hydrogel volumes absorbed similar relative amounts of polycation (Figure 5B). Important to notice that the absorption curves for all hydrogel volumes show a decrease in the molar ratio of monomer units over time after reaching a maximum value, implying a release of polycations. To confirm this, we performed an additional experiment for hydrogels with a starting volume of 57 mm^3^. Here, the hydrogels exposed to pDMAEMA-Cy3 solution and shrunken for 48 h were subsequently moved to polycation-free PBS solution for 20 days. We analyzed the supernatant at different time points and determined that the polycation release reached approximately 7.5% after 5 days of incubation in PBS and 11% after 20 days, while the hydrogels remained shrunken. The reason might be the release of an excess of polycation that could enter the hydrogel when the negative charge of the hydrogel network has not been fully compensated yet. Then, by reorganization of the complexed polymers and formation of optimal binding pairs between oppositely charged polymer chains, the weakly bound polycations were released back into the supernatant. Additionally, we demonstrated that the hydrogels exhibit only slight swelling and change in shape over the course of one and a half year in PBS 7.4 at RT. This observation demonstrates that hydrogel shrinking is very stable under physiologically relevant conditions (Figure S14).

To conclude, we demonstrated that the electrostatic interaction-based shrinking approach can be applied to objects of different sizes. However, the increase in volume of the hydrogels leads to decreased shrinking kinetics. Important to notice that a surface-to-volume ratio may change shrinking kinetics for the objects of the same volume but different shapes. In this case, shrinking will occur faster for hydrogels with a higher surface-to-volume ratio.

### 3.2 Cytocompatibility of Optimized Electrostatic Interaction-Based Shrinking

To transition from the shrinking optimization discussed above to potential tissue engineering applications, we selected ciPTECs as a targeted cell line, which is used for biofabrication of kidney proximal tubule in vitro models.^6,44^ We observed that cell adhesive properties of pure HAMA hydrogels used for the shrinking experiments described above are not sufficient for ciPTECs (Figure S15). Therefore, to facilitate cell adhesion, we modified HAMA_1500_ with RGD peptides (DoF_RGD_ = 5.8%) according to the reaction scheme shown in Figure 6. We designed a hydrogel construct with an initial volume of 290 mm^3^, featuring three parallel perfusable channels (approximately 400 μm in diameter) to facilitate the seeding of ciPTECs, thereby replicating their natural tubular environment (Figure S7). Making use of the obtained model, we aimed to evaluate the cytocompatibility of the shrinking process with the optimized above conditions (1 wt % HAMA-RGD hydrogels, 0.1 wt % pDMAEMA concentration (5 mL for 290 mm^3^ hydrogels: initial charge ratio 2.5:1) with the molecular weight ≥ 200 kDa). We compared cell viability for two different concentrations of pDMAEMA: 1 wt % (initial charge ratio of 25:1), which represents a polycation excess that facilitates rapid shrinking, and 0.1 wt % (initial charge ratio of 2.5:1), which corresponds to the minimum effective concentration for shrinking as previously established.

**Figure 6:**
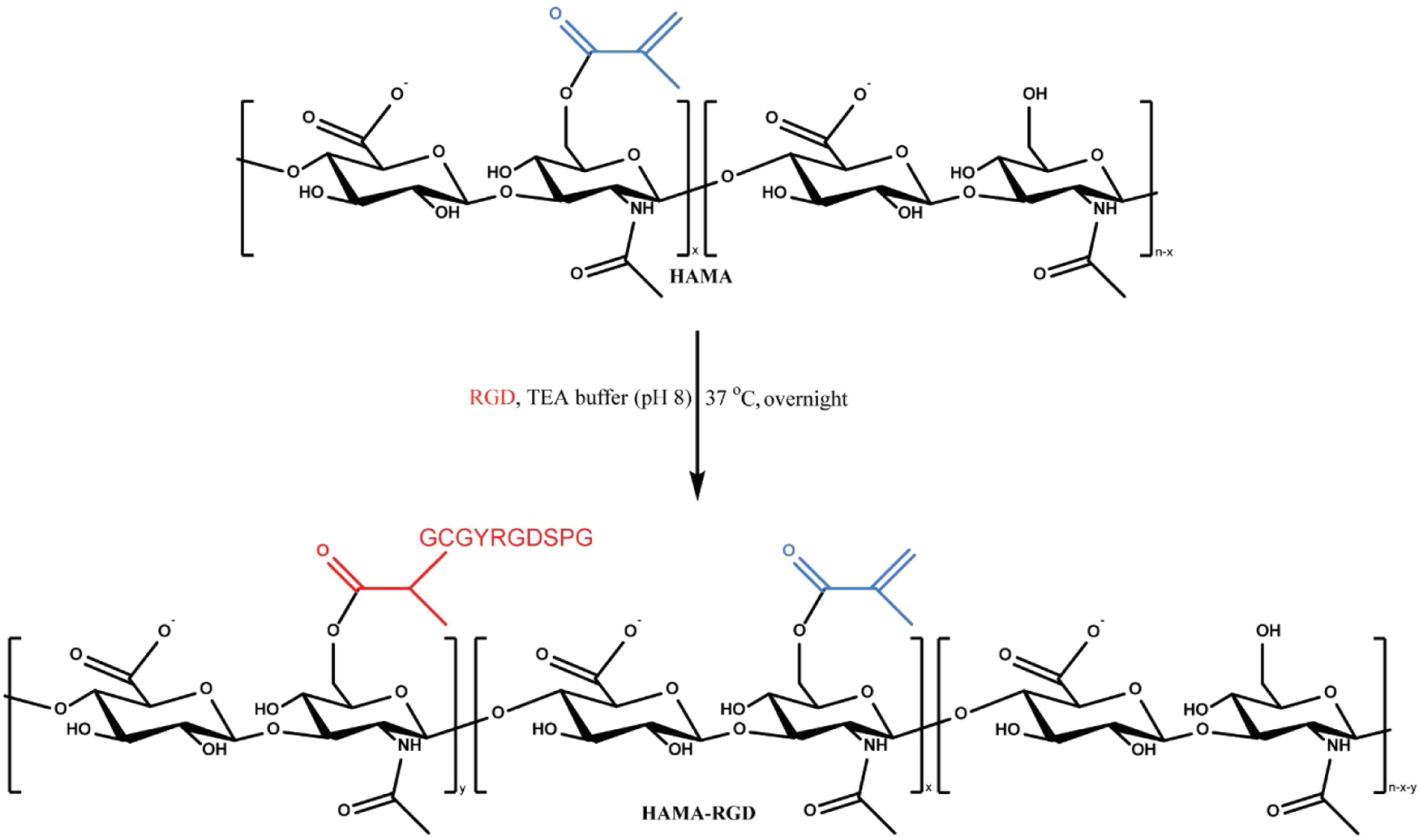
Synthesis of HAMA_1500_-RGD via Michael addition.

Shrinkage of the complete hydrogel object for both conditions was effective, as shown in Figure S16. The results of the live–dead staining of the control samples (nonshrunken) at different time points demonstrate that cells seeded into channels of HAMA-RGD hydrogels can adhere and spread over the surface of the developed material, while showing the ability to proliferate over time with the tendency to form a monolayer (Figure 7). Analysis of the images obtained for samples shrunken in pDMAEMA solutions during the first 8 days, when samples were kept at 33 °C (proliferation stage) demonstrate that cells could not survive in the hydrogels exposed to 1 wt % polycation solution (initial charge ratio 25:1) as the channels were fully red indicating the presence of only dead cells for all imaged time points. In comparison, exposing the hydrogels to 0.1 wt % of polycations (initial charge ratio 2.5:1), resulted in enhanced cell survival. However, we observed inconsistency between times points in terms of cell viability, where some samples showed high cell viability (>90% on days 1 and 8) and others displayed moderate to low viability (4–60% on day 5) as shown in Figures 7 and S17. Interestingly, imaging on day 14, when cells were kept for 6 days at 37 °C (maturation stage), demonstrated high cell loss not only for samples exposed to polycation solutions but also for control samples (Figure S18). This observation may indicate that the maturation stage induces a selection process as a result of which only part of the cells remains alive. In a previously published paper, Gong et al. demonstrated that the exposure of hydrogel constructs containing relatively robust MCF-7 breast cancer cells to 1 wt % solution of quaternized chitosan was detrimental even for a short exposure time of 4 h. Moreover, a more sensitive cell line such as human umbilical vein endothelial cells (HUVECs) demonstrated an even more pronounced decrease in cell viability.^16^ In comparison with the optimized conditions, we demonstrated that ciPTECs are able to survive even for 24 h of polycation exposure. Shrunken channels on day 8 showed a slightly nonuniform shape with deformations of the channel’s cross-section (Figure S19). However, the channels remained perfusable, and their circumference reduced almost by a factor of 2 in comparison with the control sample which translated to a final channel diameter of on average 200 μm. Concluding, the optimized shrinking conditions made the electrostatic interaction-based approach more promising in combination with cells. However, further advancements are needed to make the electrostatic interaction-based shrinking fully translatable to tissue engineering applications. Moreover, the long-term application of the in vitro models obtained through the electrostatic interaction-based shrinking approach may be limited due to potential degradation of the hydrogel matrix over time. This degradation, caused by exposure to enzymes released by cells, could lead to the release of polycations. However, as this degradation and release will be gradual, the concentrations of the released compounds are expected to remain below any relevant toxicity threshold.

**Figure 7:**
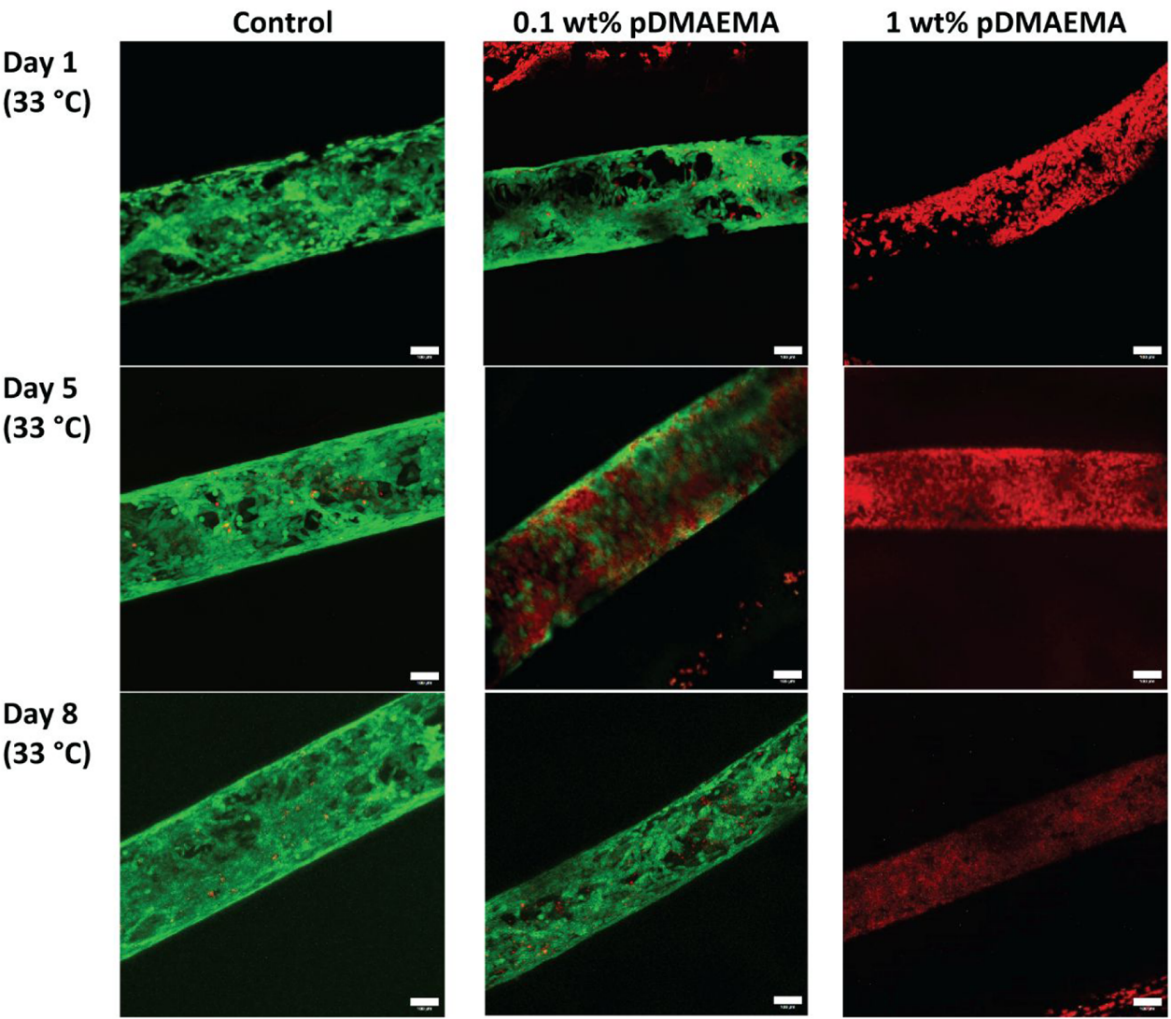
Representative confocal images (10×) of live/dead staining of ciPTECs seeded in hydrogel channels at days 1, 5, and 8 (*n* = 3 channels per time point), the scale bar is 100 μm. Two hydrogel groups were shrunken through the electrostatic interaction mechanism with 0.1 and 1 wt % pDMAEMA and compared to the untreated control group. Live cells stained in green with calcein AM (revealing calcein after hydrolysis, which is green) and dead in red with propidium iodide.

### 3.3 Electrostatic Interaction-Based Shrinking of Volumetrically Printed Hydrogels

As a proof-of-concept, we investigated the application of the shrinking technique on volumetrically printed hydrogel constructs. Volumetric printing is chosen from a wide array of 3D printing approaches given its high freedom of design, fast printing speeds (tens of seconds), and its ability to fabricate clinically relevant-sized constructs in such short times.^21,45^ Over the last years, the volumetric printing technique has been pushed toward higher resolutions. However, up until now, it was possible only with a limited material library, where the most abundant one is gelatin methacryloyl (GelMA).^46,47^ This material is promising due to its thermogelling properties and well-characterized photopolymerization that helps to prevent the sinking of the structures during printing and achieve high printing accuracy and resolution.^46^ However, printing the hydrogels with a volumetric technique at the regime that works for a broader range of materials and increasing their resolution by the presented shrinking approach is an appealing strategy to fabricate complex structures on demand with diverse properties.

In this study, we report for the first time the volumetric printing of high molecular weight hyaluronic acid-based materials alone without the addition of other polymers. To successfully volumetrically print structures from HAMA low-wt % macromer solutions, it was necessary to add 0.002 wt % of the radical inhibitor (2,2,6,6-tetramethylpiperidin-1-yl)­oxyl (TEMPO). This inhibitor captures radicals, that are highly mobile, from forming off-target cross-links outside of the volume of interest, which facilitates good printing fidelity.^48–50^

To evaluate the shrinking behavior of volumetrically printed 1 wt % HAMA structures, simple star-shaped constructs were fabricated and shrunken in a buffer solution of pDADMAC (400–500 kDa) as a proof-of-concept. After 24 h of shrinkage, the whole volume of the hydrogel star was significantly decreased, while the shape was slightly changed. The tips decreased their size more than the main body and had a width of 42 ± 6 μm while they originally had widths on the order of 450 μm (Figure 8A,B). We attribute this nonuniform shrinking to the slow polycation diffusion and resulting inhomogeneous distribution within a hydrogel structure at the time of imaging. This effect was also observed for a small disc model used for kinetics characterization, where the complete and uniform shrinking was observed only after 48 h (Figure 4A,C). The remarkable reduction in spatial dimensions of the star-shaped hydrogel represents one of the smallest printed features so far reported for volumetric printing.^46–48^ To compare, in a recent publication by Falandt et al., it was demonstrated to resolve a ∼24 μm-wide feature as part of a larger printed object.^47^ However, as mentioned above, this feature size was achieved when using gelatin-based materials, and by combining shrinking technology with volumetric printing we demonstrated that it is possible to achieve comparable features for HAMA-based materials.

**Figure 8:**
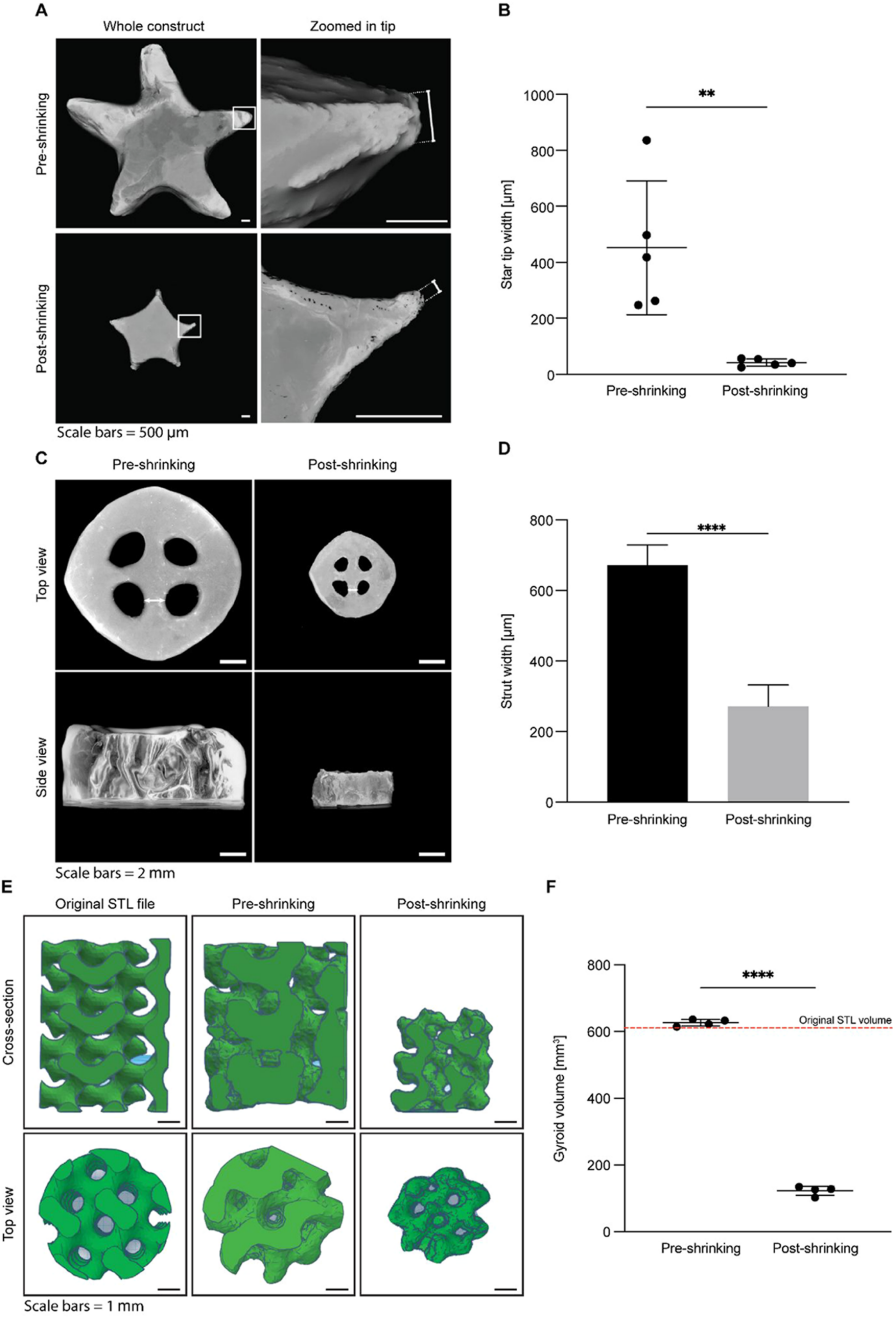
(A) Zoomed-in microcomputed tomography (μCT) images of original and shrunken volumetrically printed stars made from 1 wt % HAMA. (B) The smallest feature sizes of the volumetrically printed stars before and after shrinking (*n* = 5 for all data points). (C) Representative volumetrically printed perforated hydrogel disc made from 1 wt % HAMA before and after shrinking. (D) Quantified spoke widths of the printed disk object before and after shrinking. (E) A volumetrically printed hydrogel gyroid made from 4 wt % GelMA B before and after shrinking, visualized using μCT. (F) Measured volumes of the gyroid structure before and after shrinking. All objects were shrunken for 24 h in a 2 wt % pDADMAC solution (RT, pH 7.4, ionic strength 170 mM, polymer molecular weight 400–500 kDa).

Next, a more complex structure, namely, a hollow hydrogel disc with a wavy wall insert was printed. Again, a 1.0 wt % HAMA macromer solution was used and the object was subsequently shrunken by exposure to pDADMAC (400–500 kDa) (Figure 8C,D). A uniform shrinking of this printed structure resulted in a ∼9× reduction in volume after the water was expulsed, which is in agreement with the shrinking factors obtained in the previous sections for the bulk cylindrical hydrogels. Furthermore, measurements on the internal spoke widths of the hydrogels showed that the initial width of 770 ± 61 μm was reduced by a factor of 2.6 to 301 ± 50 μm after shrinking (Figure 8D).

To evaluate whether postprocessing of printed objects is compatible with more intricate structures, which can be challenging to print using conventional additive manufacturing techniques, a gyroid design was volumetrically printed using a 4.0 wt % GelMA B solution (Figure 8E,F). Gelatin was chosen because, as mentioned earlier, it allows for easier printing of more complex structures. Moreover, gelatin-based hydrogels also present a negatively charged network that can be shrunken by exposure to polycations.^16^ Again, the water expulsion-based postprocessing technique proved to be excellently capable of decreasing the minimal obtainable printing feature size by reducing the initial printed volume 5 times while retaining the important structural features. The miniaturization of the various printed structures presented here demonstrates the potential for incorporating postprocessing methods into volumetric 3D printing by shrinking printed hydrogels through water expulsion.

## 4 Conclusions

In this study, we demonstrated the power of electrostatic interaction-based hydrogel shrinking and elucidated the important parameters contributing to the shrinking kinetics and overall shrinking factor. We demonstrated that HAMA molecular weight and its DM did not affect the shrinking factor, while it was important to keep the initial HAMA content in hydrogels low (1 wt %) to achieve high shrinking values. In turn, the polycation parameters, molecular weight, and concentration showed substantial influence on the shrinking process. The most efficient shrinkage was achieved when the molecular weight of the polycation was at least 200 kDa. Simultaneously, experiments varying polycation concentrations, which correspond to changes in the system’s charge ratio, demonstrated that the initial charge ratio of positively to negatively charged groups should be at least 2.5:1. The shrinking experiments with the hydrogels containing ciPTECs using optimized conditions showed improved cytocompatibility, potentially paving the way for this technique to be applied in biofabrication. Combining the shrinking approach with volumetric printing showed a pronounced resolution enhancement of the printed hydrogel structures with the achieved feature size of 42 ± 6 μm, one of the smallest reported for volumetric printing so far. Moreover, the enhanced resolution of the gyroid structure (a model of a complex convoluted tissue) after shrinking showed the promise of a volumetric printing and shrinking strategy for high-resolution tissue biofabrication. In the future, such 3D-printed hydrogels could potentially be used to develop in vitro models for drug testing or scaffolds for tissue regeneration with physiologically relevant sizes mimicking in vivo conditions.

## Supplementary Material



## Notes

Additional experimental details on the characterizations of the synthesized polymers and the shrinking optimizations for both normal and cell-seeded hydrogels (PDF)
