# Supplementary material for: In-Depth Investigation of Electrostatic Interaction-Based Hydrogel Shrinking for Volumetric Printing and Tissue Engineering Applications
Source: Biomacromolecules. 2025 Jun 16;26(7):4108–23. doi: 10.1021/acs.biomac.5c00117 (PMC12273623; doi:10.1021/acs.biomac.5c00117)
Supplement: Supplementary file 1 [file bm5c00117_si_001.pdf]

Supporting information: In-depth investigation of electrostatic interaction-based hydrogel shrinking for 3D printing and tissue engineering applications

Dmitrii Iudin, Léon J.J.A. Gerridzen, Paulina N. Bernal, Carl C.L. Schuurmans, Myriam Neumann, Lam Nguyen, Mies J. van Steenbergen, Jaimie Hak, Wanlu Li, Cristina Casadidio, Anne Metje van Genderen, Rosalinde Masereeuw, Riccardo Levato, Yu Shrike Zhang, Bas G.P. van Ravensteijn and Tina Vermonden

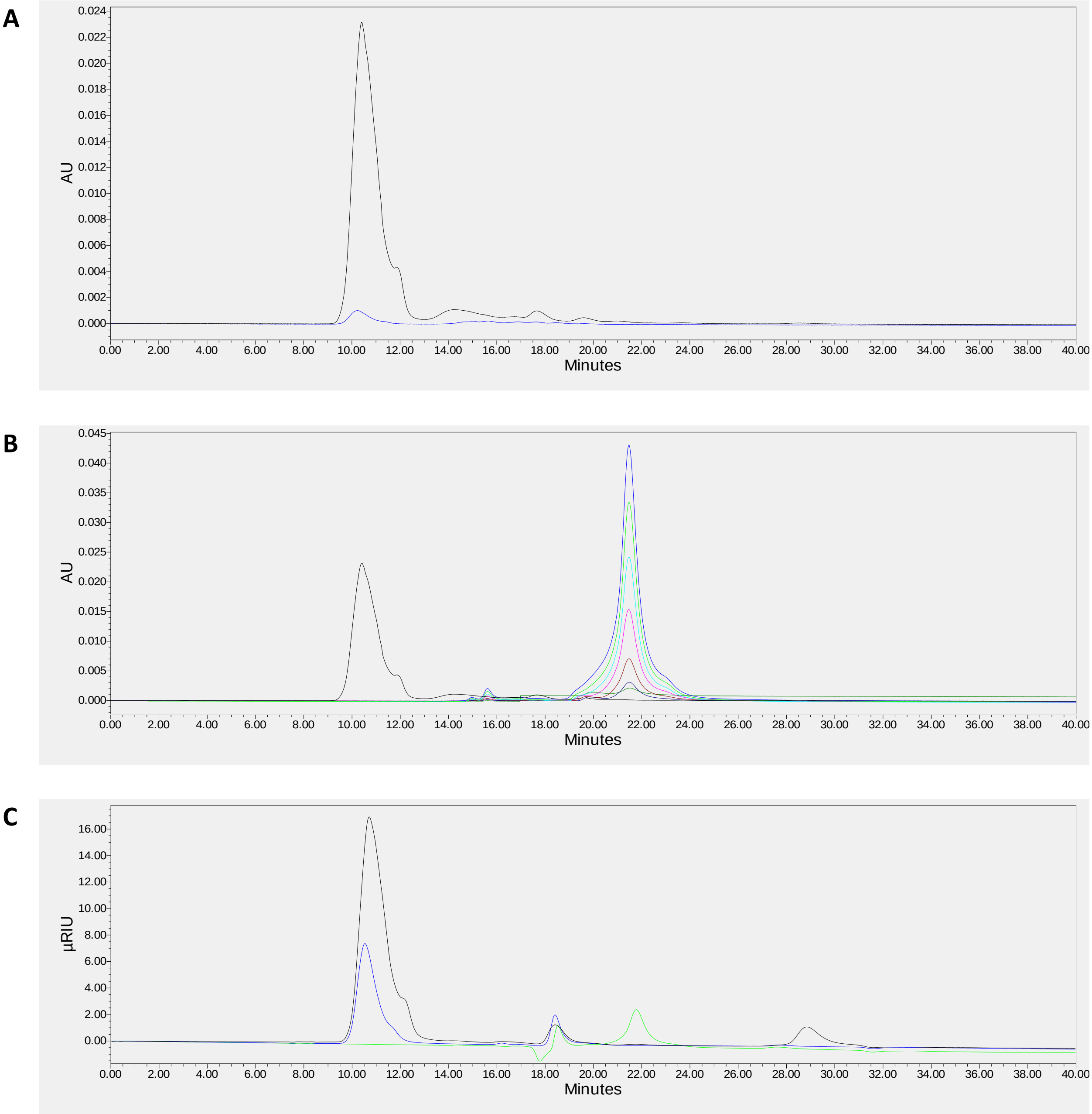

Figure S1. SEC analysis, UV detector (275 nm) **A:** black – HAMA-RGD, blue – HAMA; **B:** black signal at 10.5 min – HAMA-RGD, other signals at 21.5 min – free RGD calibration 0.05 – 1 mg/mL. SEC analysis, RI detector **C:** black – HAMA-RGD, blue – HAMA, green – RGD.

Calculations of DoF_{RGD}: 1) amount of RGD in HAMA-RGD sample was determined as 0.59 mg/mL, the starting concentration of HAMA-RGD sample was 5 mg/mL. 2) % of RGD in HAMA-

RGD is (0.59 mg / 5 mg) × 100% = 11.8%. 3) % of HAMA in HAMA-RGD then (100 – 11.8)% = 88.2%. 4) Molecular weight of HAMA-RGD unit (p): (M(HAMA) / p) × 100% = 88.2% where M(HAMA) is 417 g/mol for the disaccharide unit then p = 472.8 g/mol. 5) DoF_{RGD} (y): M(HAMA) + y × M(RGD) = 472.8 g/mol where M(RGD) is 968 g/mol then y = 0.058 or 5.8%.

A

B

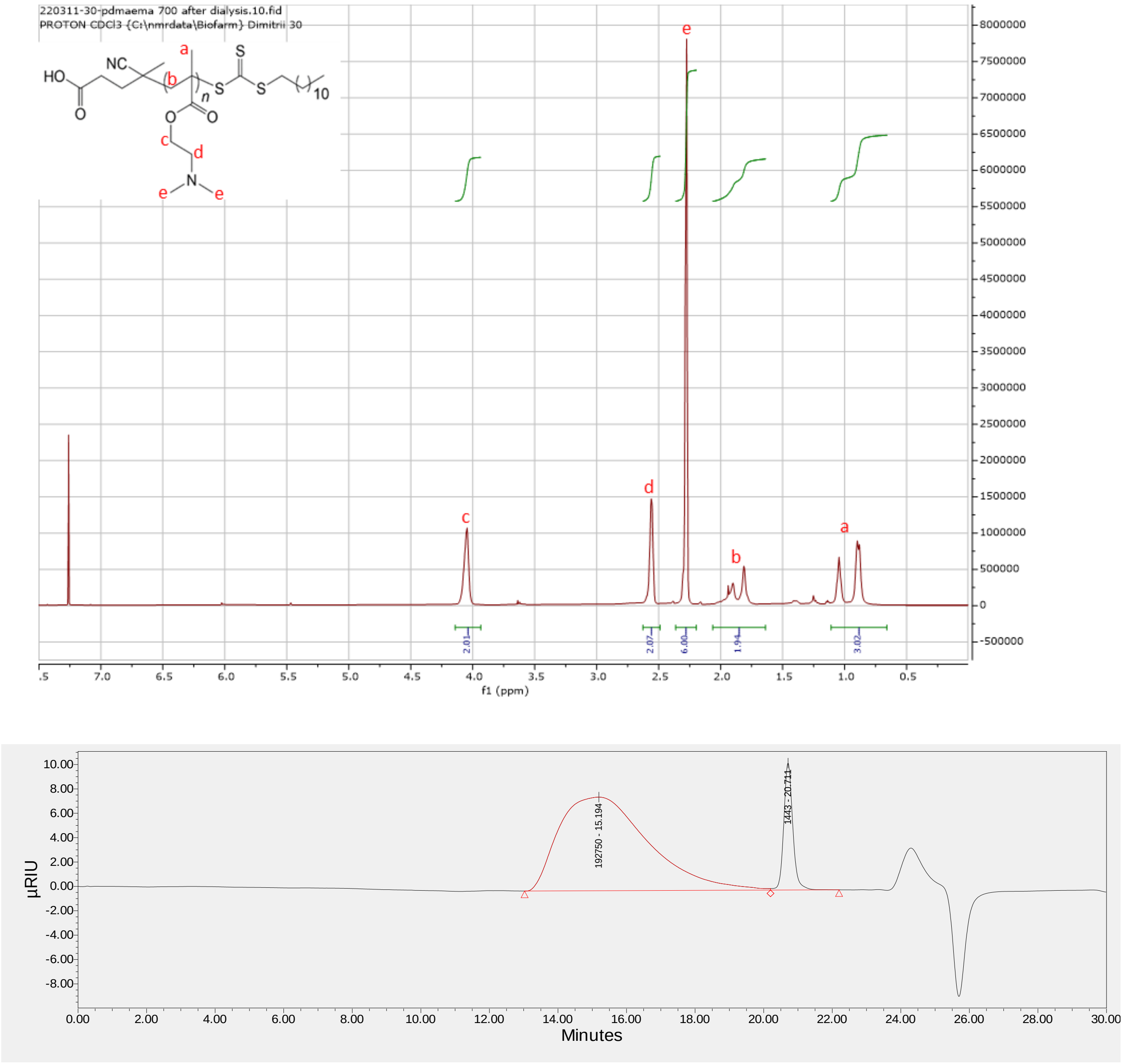

Figure S2. pDMAEMA: ^{1}H-NMR spectrum in $CDCl_3$ (**A**) and SEC analysis (**B**).

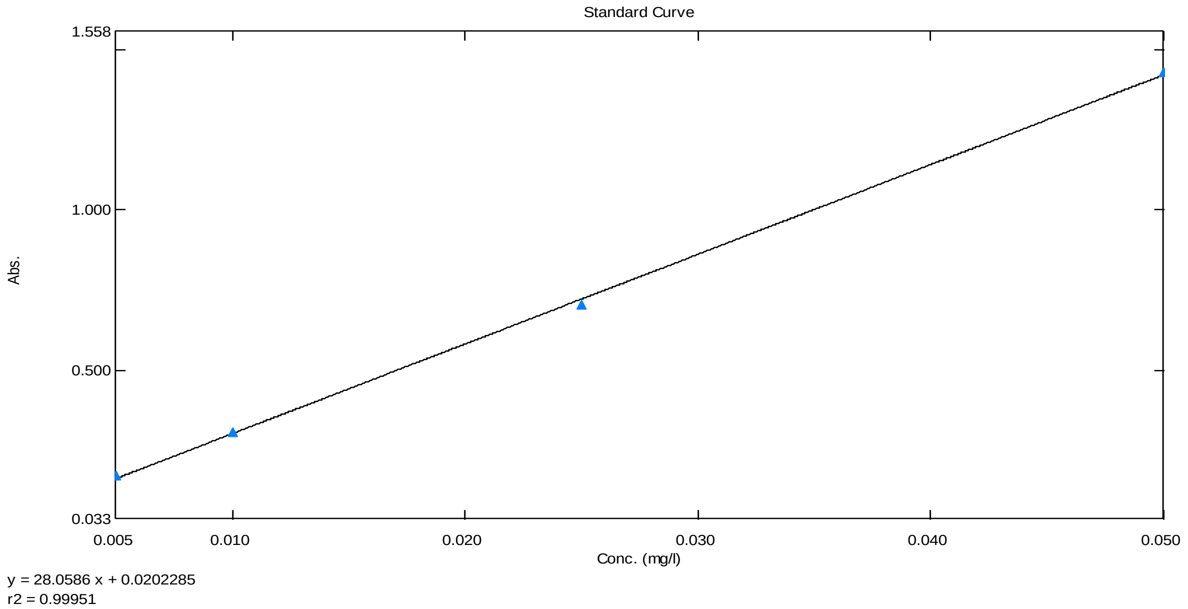

Figure S3. Calibration curve of 4-cyano-4-[(dodecylsulfanylthiocarbonyl)sulfanyl]pentanoic (CTA). Calculation of amount of pDMAEMA containing CTA chain ends: 10.5 mg/ml of pDMAEMA sample contains 0,026 mg/ml of CTA chain ends according to calibration curve. $n_{pDMAEMA} = m_{pDMAEMA}/Mn_{pDMAEMA}$ = 10.5 mg / 116700 g/mol = 9×10^{-5} mmol and n_{CTA} = 0.026 mg / 403.67 g/mol = 6.4×10^{-5} mmol. Then, $\%_{pDMAEMA\text{-}CTA} = 6.4\times10^{-5}/9*10^{-5} \times 100\% = 71\%$.

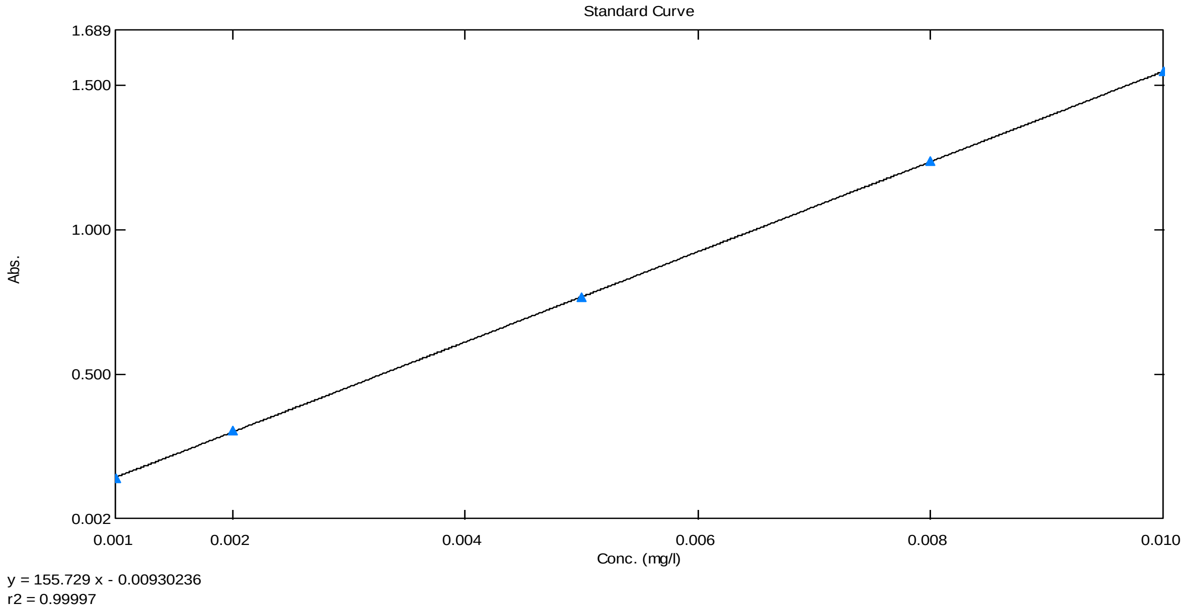

Figure S4. Calibration curve of Cy3-maleimide dye. Calculation of the coupling efficiency of Cy3: 10 mg/ml of pDMAEMA-cy3 sample contains 0,004 mg/ml of cyanine dye according to calibration curve. $n_{pDMAEMA}$ = 10 mg / 116700 g/mol = 8.6×10^{-5} mmol and n_{Cy3} = 0.004 mg / 666.56 g/mol = 6×10^{-6} mmol. Then, $\%_{pDMAEMA\text{-}Cy3} = 0.6\times10^{-5}/8.6\times10^{-5} \times 100\% = 7\%$, but considering that only 71% of polymers had CTA chain end available for modification after previous step then coupling efficiency was 10%.

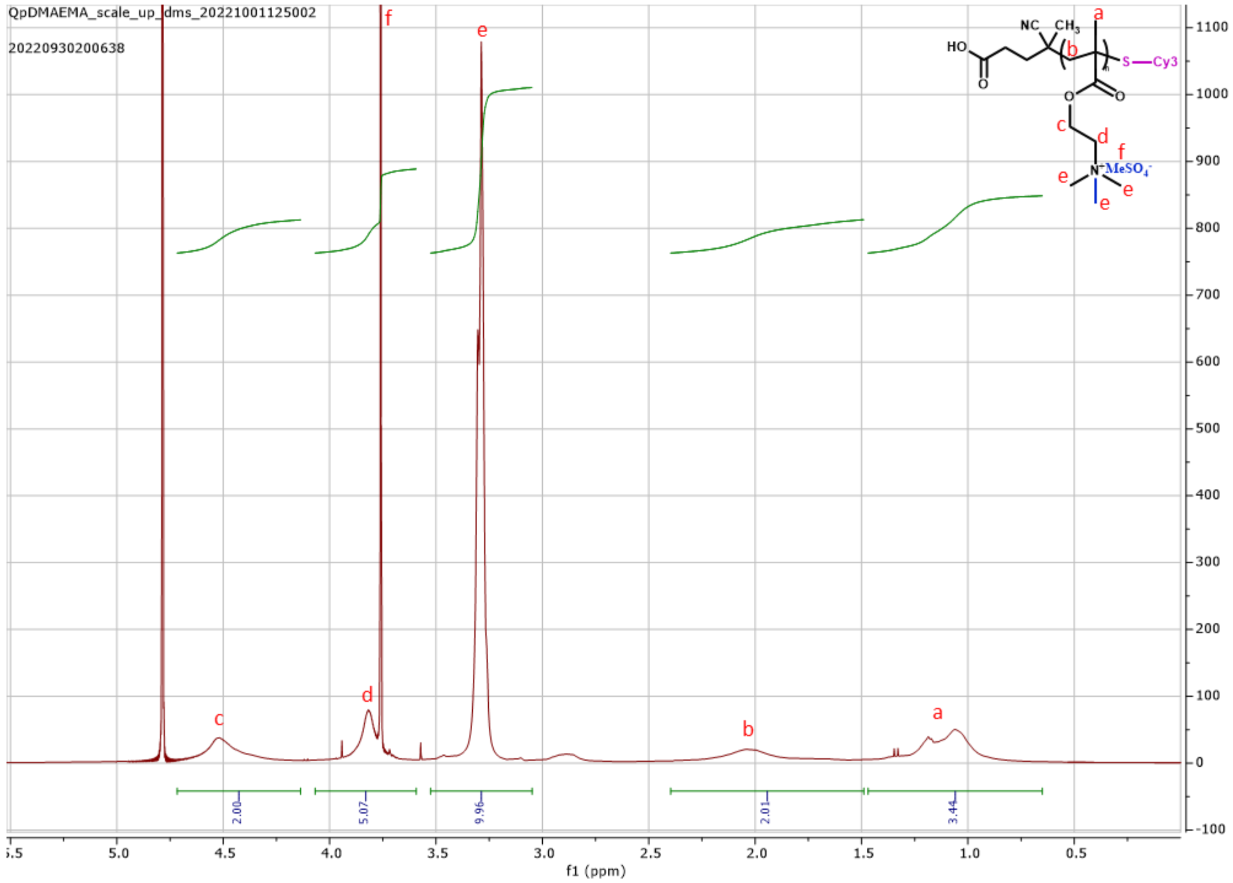

Figure S5. ^{1}H-NMR spectrum of QpDMAEMA-Cy3 in D_2O.

A

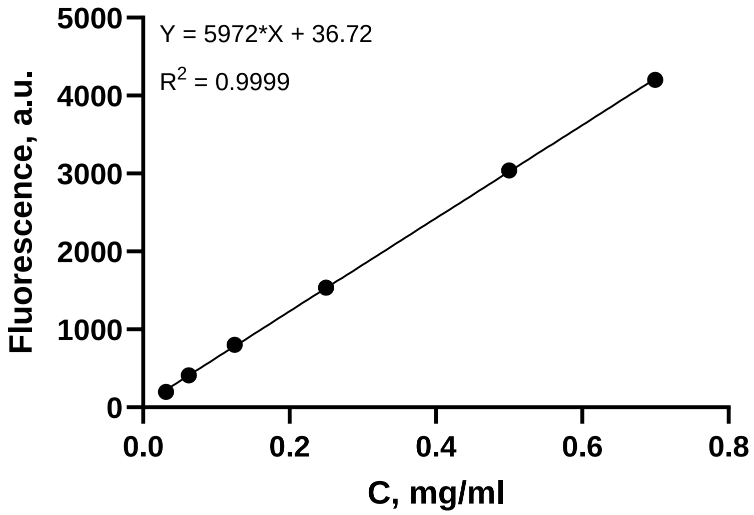

B

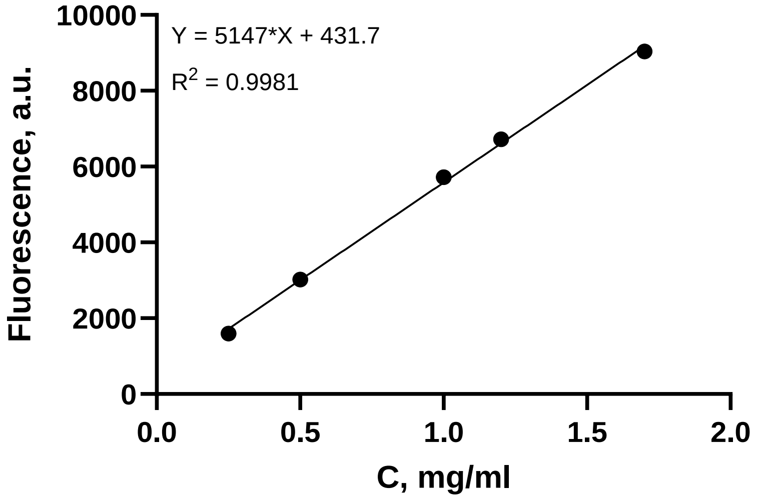

C

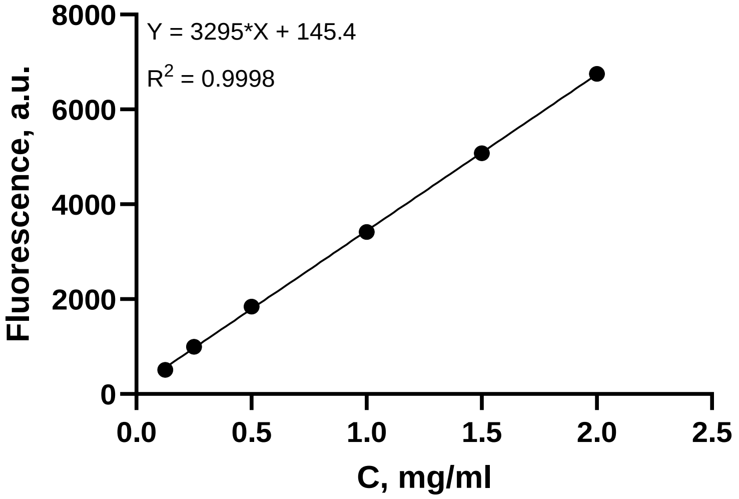

Figure S6. Calibration curves for pDAEMA-Cy3 in the range of 0.03125 – 0.7 mg/mL (**A**), 0.25 – 1.7 mg/mL (**B**) and for QpDMAEMA-Cy3 0.125 – 2 mg/mL (**C**).

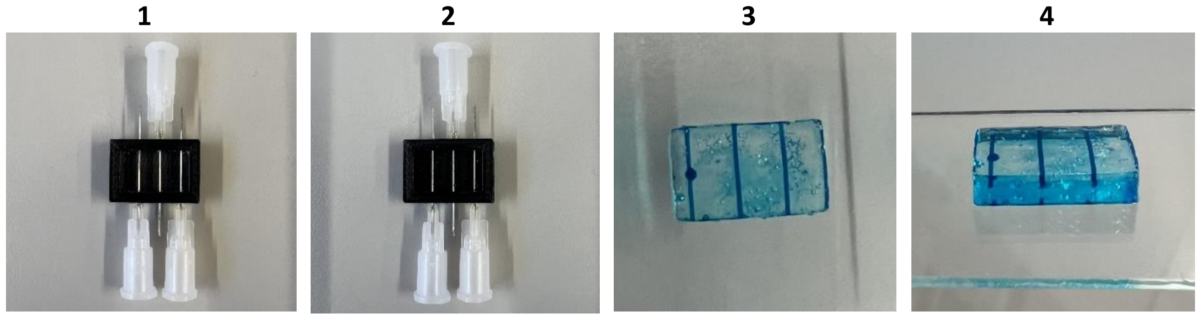

Figure S7. The pictures of the set-up to prepare the hydrogel model with perfusable channels. The hydrogels were obtained by using a plastic rectangular mold (length: 12 mm, width: 8 mm, height: 3 mm, the final volume ~ 290 mm^3). **Picture 1** - empty mold with three inserted 27G needles; **Picture 2** - mold filled-in with polymer solution; **Picture 3** - crosslinked hydrogel perfused with Alcian blue (top view); **Picture 4** - crosslinked hydrogel perfused with Alcian blue (side view).

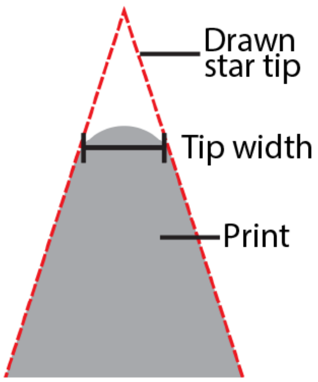

Figure S8. Diagram showing the measurement approach for volumetrically printed star tip widths.

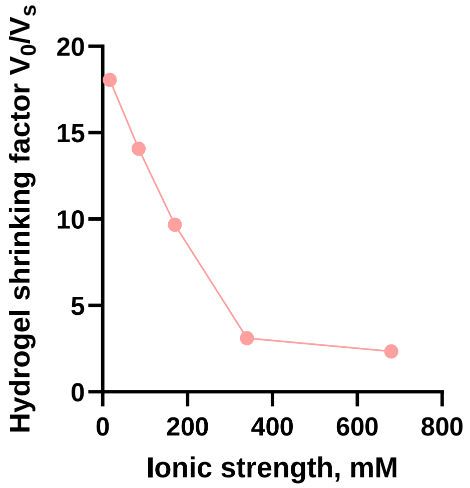

Figure S9. Shrinking factors for 1.0 wt% HAMA hydrogels upon incubation with solutions of pDADMAC with Mw 400-500 kDa as a function of the ionic strength of the buffer solutions at pH 7.4. Data are shown as the average of 3 samples ± SD, for all time points deviation bars are too small to be visible.

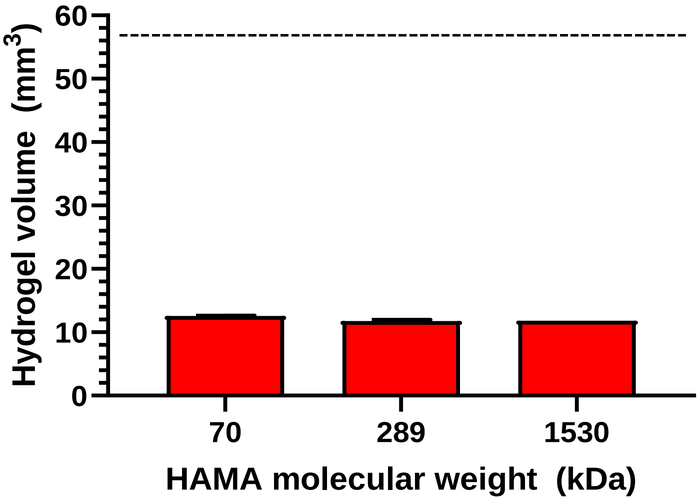

Figure S10. Shrunken 1.0 wt% HAMA hydrogel volumes after incubation until shrinking equilibrium was reached in an 85-mM ionic strength, pH 7.4 PB solution at room temperature supplemented with 2.0 wt% pDADMAC (Mw < 100 kDa). Hydrogel volume after photopolymerisation (V_0) was 57 mm^3 (dashed line). HAMA macromer initial molecular weight was ~70, 289, or 1470 – 1530 kDa and the DM was approximatelly 23 %. Data are shown as the average of 3 samples ± SD.

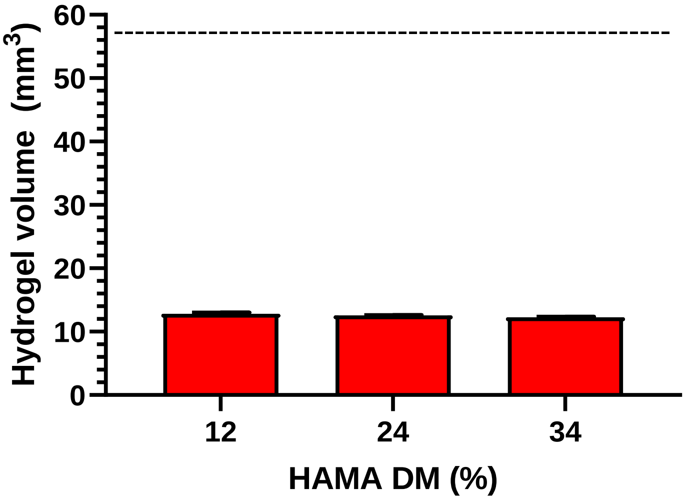

Figure S11. 1.0 wt% HAMA hydrogel volumes after incubation until shrinking equilibrium was reached in an 85-mM ionic strength, pH 7.4 PB solution at room temperature supplemented with 2.0 wt% pDADMAC (Mw < 100 kDa). Initial hydrogel volume (V_0) was 57 mm^3 (dashed line). HAMA macromer initial molecular weight was ~70 kDa and DM was 12, 24, or 34 ± 3%. Data are shown as the average of 3 samples ± SD.

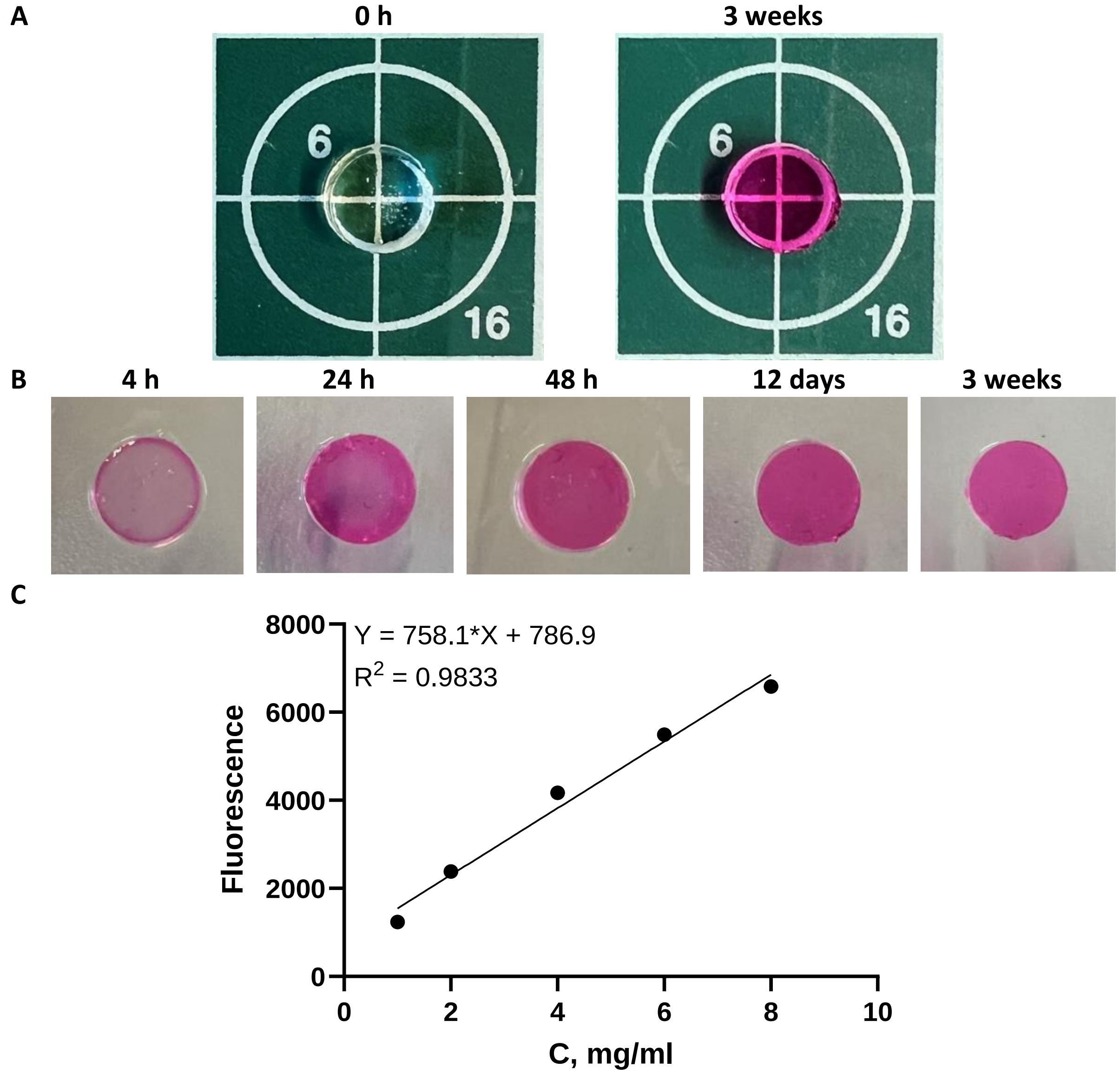

Figure S12. A: 8 wt% $HAMA_{70}$ hydrogel placed in 0.8 wt% pDMAEMA-Cy3 soltuion for 3 weeks with no significant shrinking observed. **B:** Macroscopic pictures of pDMAEMA-cy3 uptake and distribution in 8 wt% $HAMA_{70}$ hydrogels over time. **C:** Calibration curve of pDMAEMA-Cy3 in the range of 1 – 8 mg/mL. Based on this calibration curve the supernatant of the shrinking sample was analysed and the charge ratio inside the hydrogel was estimated approx. 1:1, which is aroud theoretical charge neutralization.

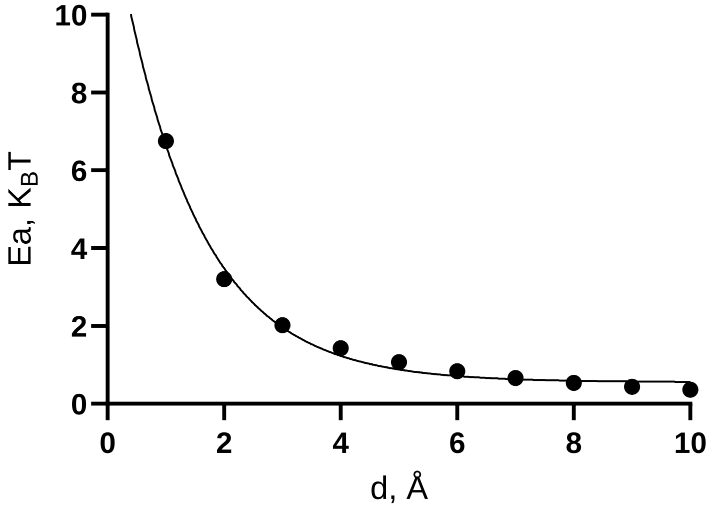

Figure S13. Graph of the complexation strenght of an ion pair expressed in K_BT depending on d – the contact distance of the charged groups in an ion pair expressed in Å (calculations based on the equation published in literature)[1]. In our experiments, the following molecular weights of pDADMAC were used <100, 200 – 350 and 400 – 500 kDa with an avarage amount of monomer units <600, 1200 – 2150 and 2500 – 3100. The complexation strength with the values above 30 K_BT are considered to be very strong not to dissosiate at low salt concentrations (five ionic bonds with 6 K_BT per bond).[1] Therefore, even for the shortest polycations used here the strength of the electrostatic interactions is higher than 30 K_BT.

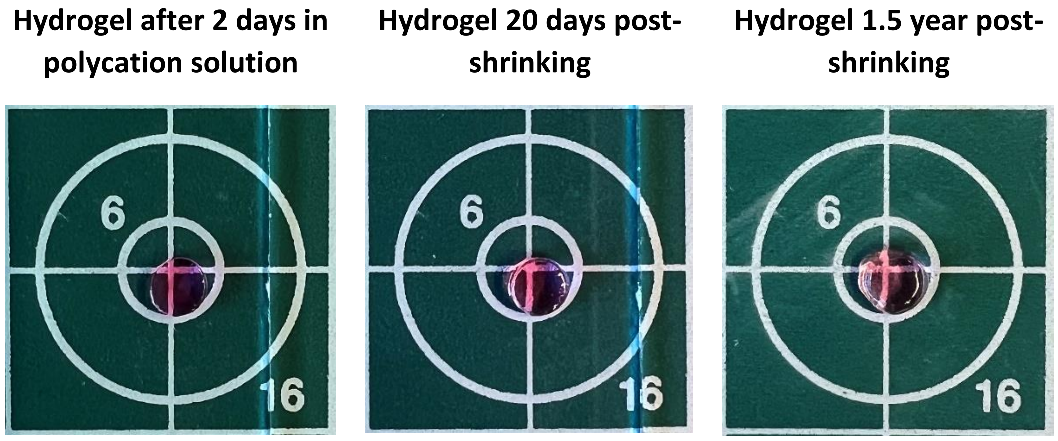

Figure S14. 1 wt% HAMA hydrogel shrunken with pDMAEMA-Cy3 (6.4 µmol/mL of monomer units) for 2 days and incubated in polycation-free PBS 7.4 for 20 days and 1.5 year. The starting diameter of the hydrogel was 6 mm (the inner white circle).

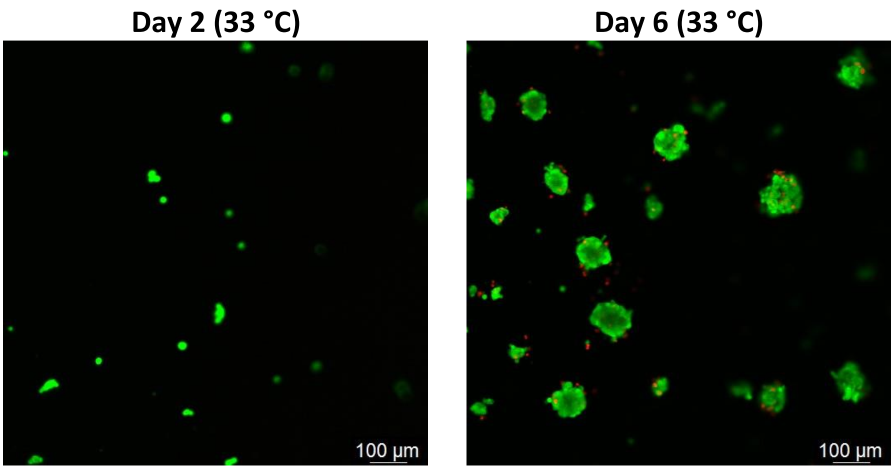

Figure S15. Confocal images (10×) of live/dead staining of ciPTECs seeded on 1 wt% $HAMA_{1500}$ hydrogel discs (n = 3), the scale bar is 100 µm. Live cells stained in green with calcein AM and dead in red with propidium iodide.

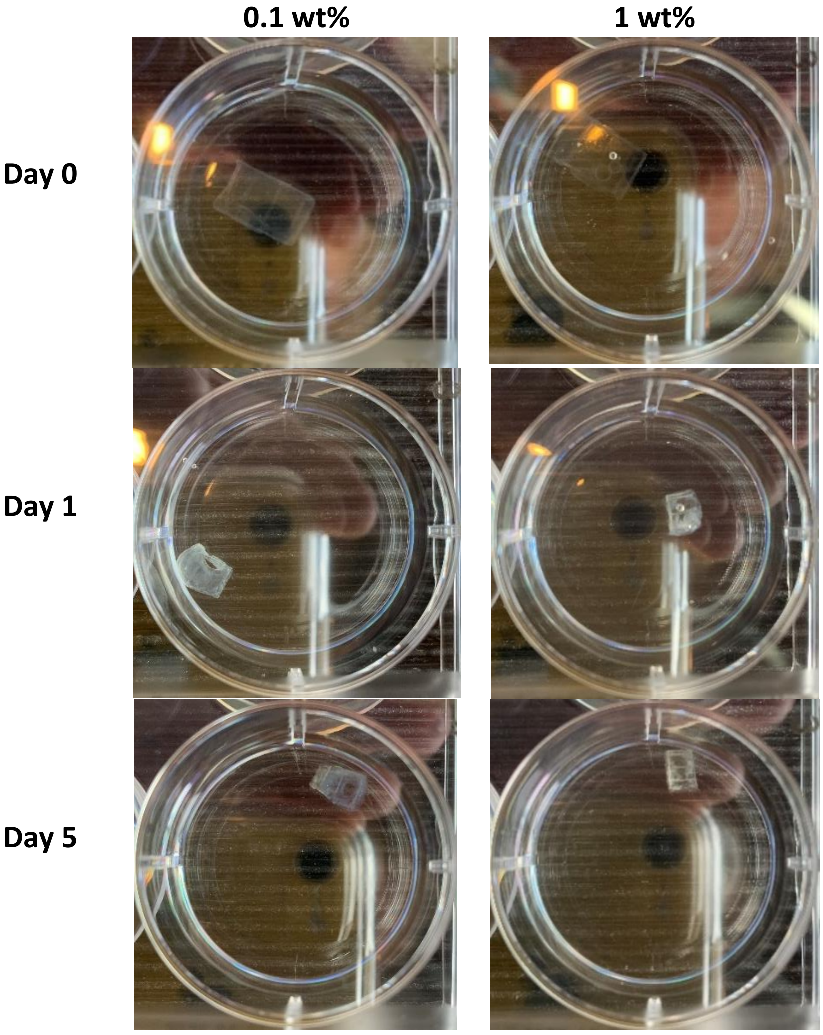

Figure S16. Macroscopic pictures of hydrogel shrinking with 0.1 and 1 wt% solutions of pDMAEMA at time points 0, 1 and 5 days.

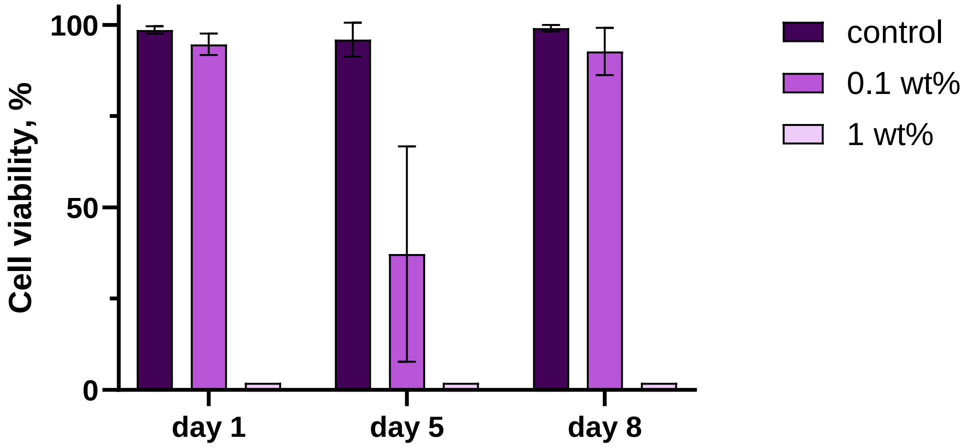

Figure S17. Semi-quantitative analysis of ciPTECs viability in hydrogel channels on day 1, 5 and 8 (n = 2 – 3 channels per day). Two hydrogel groups were shrunken through electrostatic interactions mechanism with 0.1 and 1 wt% pDMAEMA and compared to the untreated control group. The analysis was performed by determining the coverage percentage of the channels separatelly with alive and dead cells, calculating the total coverage with both species and determining the fraction percentage of the covarage with alive cells. For that, all images obtained were processed using ImageJ: z-projections were made for half of a channel, converted to eight-bit, the thresholding was applied and the percentage of the coverage area was calculated.

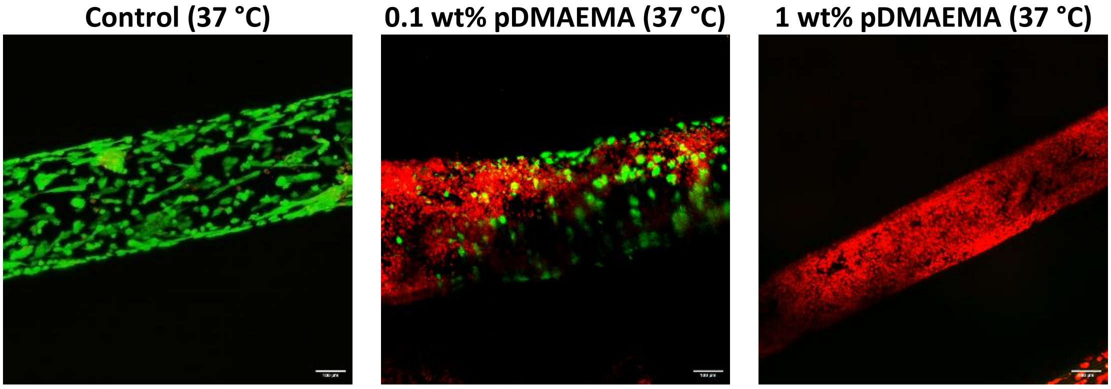

Figure S18. Representative confocal images (10×) of live/dead staining of ciPTECs seeded in hydrogel channels on day 14 (n = 3 channels), the scale bar is 100 µm. Two hydrogel groups were shrunken through electrostatic interactions mechanism with 0.1 and 1 wt% pDMAEMA and compared to the untreated control group. Live cells stained in green with calcein AM (revealing calcein after hydrolysis, which is green) and dead in red with propidium iodide.

A

B

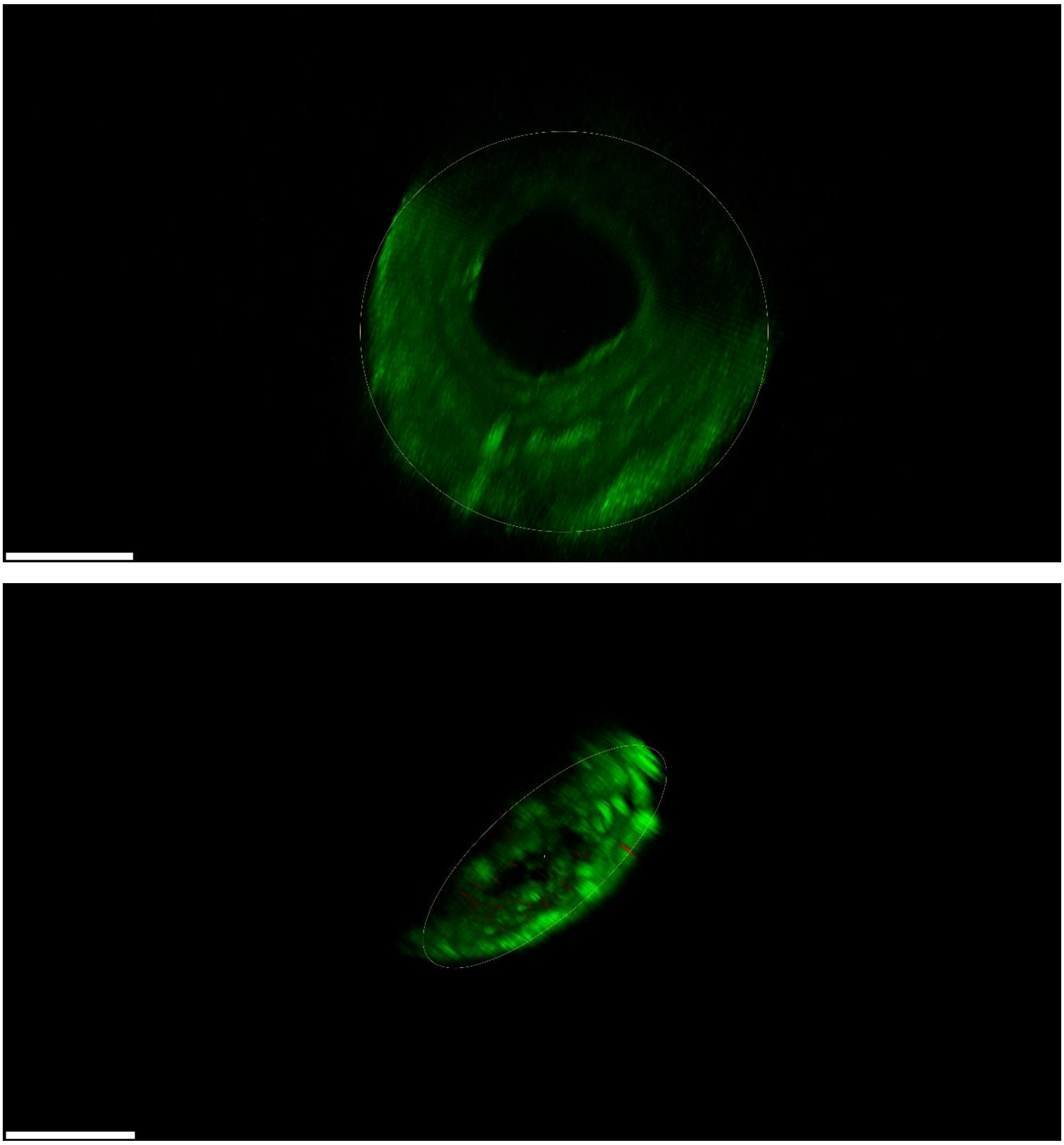

Figure S19. 3D inlet views of the channels seeded with ciPTECs on day 8 for **A:** control sample and **B:** hydrogel shrunken in 0.1 wt% of pDMAEMA-cy3. The scale bar is 200 µm. For the control sample, one side of the channel is less visible due to the confocal depth imaging limitations.

References

(1) Spruijt, E.; van den Berg, S. A.; Cohen Stuart, M. A.; van der Gucht, J. Direct Measurement of the Strength of Single Ionic Bonds between Hydrated Charges. *ACS Nano* **2012**, *6* (6), 5297–5303. https://doi.org/10.1021/nn301097y.
